# Unlocking the Potential of Poly(*Ortho* Ester)s: A General Catalytic Approach to the Synthesis of Surface‐Erodible Materials

**DOI:** 10.1002/anie.201709934

**Published:** 2017-12-04

**Authors:** Mathieu J.‐L. Tschan, Nga Sze Ieong, Richard Todd, Jack Everson, Andrew P. Dove

**Affiliations:** ^1^ Department of Chemistry The University of Warwick Coventry CV4 7AL UK

**Keywords:** olefin isomerization, poly(*ortho* ester)s, step-growth polymerization, vinyl acetal monomer

## Abstract

Poly(*ortho* ester)s (POEs) are well‐known for their surface‐eroding properties and hence present unique opportunities for controlled‐release and tissue‐engineering applications. Their development and wide‐spread investigation has, however, been severely limited by challenging synthetic requirements that incorporate unstable intermediates and are therefore highly irreproducible. Herein, the first catalytic method for the synthesis of POEs using air‐ and moisture‐stable vinyl acetal precursors is presented. The synthesis of a range of POE structures is demonstrated, including those that are extremely difficult to achieve by other synthetic methods. Furthermore, application of this chemistry permits efficient installation of functional groups through *ortho* ester linkages on an aliphatic polycarbonate.

The use of biodegradable polymers for controlled drug release and tissue engineering represents one of the most important advances in biomedicine.^1^, ^2^, ^3^ An ideal material would display a surface erosion profile in which hydrolysis occurs faster than water ingress into the materials and results in sequential erosion of the surface layers.^4^, ^5^ Such profiles enable idealized zero‐order release profiles and predictable materials properties throughout degradation.^6^ Despite these benchmark requirements, the paucity of easily accessible surface eroding materials have led to extensive study of bulk eroding materials, such as poly(lactic acid) and poly(*ϵ*‐caprolactone),^7^, ^8^ in which water diffusion into the material occurs at a comparable or faster rate to hydrolysis. This results in a non‐linear mass loss over time, amplified by autocatalysis from trapped degradation products, which in turn leads to non‐linear release by encapsulants, significant burst effects, and uncontrolled loss of mechanical stability.

Despite the clear potential advantages of surface erodible polymers, examples are limited to only a few families, such as poly(anhydride)s^9^, ^10^ and poly(*ortho* ester)s (POEs).^11^, ^12^ The milder degradation products present POEs as a potentially more attractive choice for in vivo applications and, indeed, POE types III and IV (Figure 1) have shown significant promise in ocular^11^, ^12^, ^13^, ^14^ as well as gene delivery.^11^, ^12^ However, their synthesis typically involves either step‐growth polymerization of the highly air‐ and moisture‐sensitive diketene acetal (3,9‐bis(ethylidene‐2,4,8,10‐tetraoxaspiro[5,5]undecane (DETSOU; Scheme 1) with a diol,^15^ or transesterification between a triorthoester and triol.^16^ Both procedures present significant synthetic challenges when it comes to producing repeatable polymer characteristics, which likely results because the highly reactive ketene acetal monomers compromises the high levels of purity required for successful step‐growth polymerization. More recently, some success was achieved by preparing *ortho*‐ester‐containing monomers by multistep syntheses to create poly(*ortho* ester amide)s and poly(*ortho* ester urethane)s.^17^ Despite these advances, further development of general routes to POE‐based materials is required to enable wider study; in particular, techniques are needed to overcome challenges associated with the synthesis of such materials.

**Figure 1:**
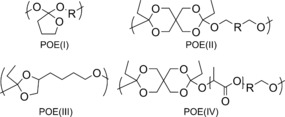
Types of poly(*ortho* ester)s (POEs).

**Scheme 1:**
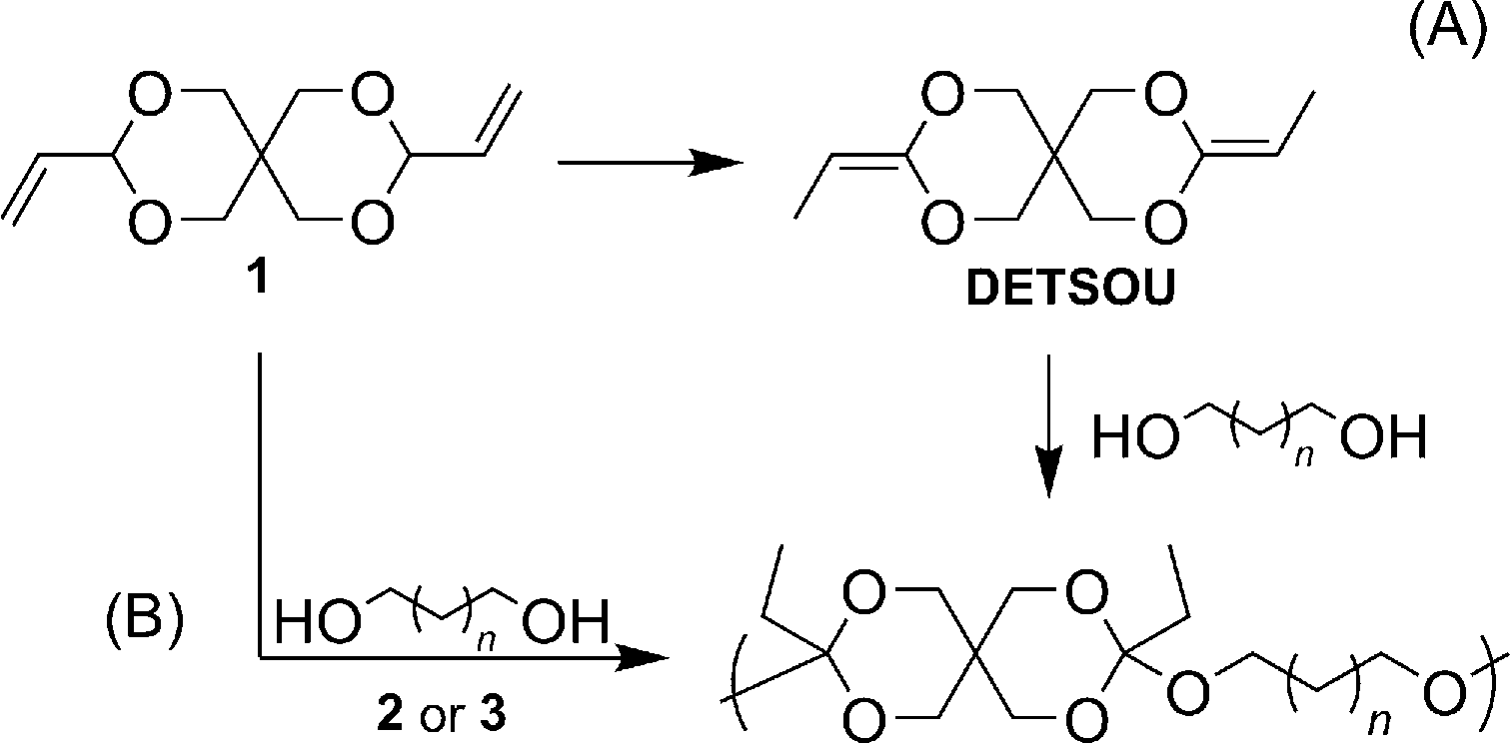
Synthesis of POE(II) from **1** and diols A) via DETSOU; B) by catalytic synthesis (**2**=[RuHCOCl(PPh_3_)_3_]; **3**=[RuHCl(PPh_3_)_3_]).

Herein, we present a facile, catalytic method for the synthesis of POEs via easy‐to‐access, air‐ and moisture‐stable intermediates. Furthermore, we demonstrate that this unique approach is the only general synthetic pathway that can yield POEs of types II, III, and IV. Moreover, the procedure is amenable to a large range of potential feedstocks, as well as to functionalization of other materials. The development of a more general and simple synthetic method to access POEs will more readily enable wider study and hence potentially address some of the outstanding problems that face the biomedical industry, including enhancing control over drug release rates and increasing the efficiency of delivery systems.

Inspired by the wealth of ruthenium‐based double‐bond‐migration catalysis in the literature,^18^, ^19^ including for the synthesis of ketene acetals,^20^, ^21^ we postulated that a 1,3‐dihydride shift of a vinyl acetal with catalysts such as [RuHCOCl(PPh_3_)_3_] (**2**)^20^ and [RuHCl(PPh_3_)_3_]^18^ (**3**) would enable in situ olefin isomerization catalysts in the presence of alcohols without the need to isolate the highly sensitive ketene acetal intermediate. Initially, model reactions focused on the in situ isomerization of the commercially available 5,5‐dimethyl‐2‐vinyl‐1,3‐dioxane, thus avoiding isolation of the highly reactive DETSOU monomer (Scheme 1). With excess 1,6‐hexanediol it was found that, while **2** catalyzed the formation of the diorthoester to 99.7 % conversion at 45 °C in 6.5 h, using an analogous catalyst loading of **3** only gave similar conversion at the same rate when carried out at 85 °C (Supporting Information). Moreover, no side products/reactions could be observed by ^1^H and ^13^C NMR spectroscopic analysis of the model reaction crude mixture, and only the expected product was formed.

The optimal conditions found for each catalyst in the model reaction were then applied to the step‐growth polymerization of the difunctional monomers **1** and 1,6‐hexandiol (Scheme 1). Notably, **1** is obtained in a straightforward one‐step reaction in 56 % yield while DETSOU was obtained by a difficult two‐step synthetic procedure in an approximately 40 % overall yield.^15^ Initially, the polymerization was attempted using catalyst **2**. While **2** was active at a lower temperature and hence limited the chance of polymer degradation, only oligomers were isolated (<1 kDa as determined by size‐exclusion chromatography (SEC) analysis in CHCl_3_). Interestingly, POE(II) of significantly higher molecular weight was only achieved when catalyst **3** was employed at an increased temperature. Monitoring the reaction by SEC analysis revealed that the molecular weight of the polymer plateaued after about 4 h, reaching a weight‐averaged molecular weight (*M*_w_) of 9.5 kDa (Supporting Information, Figure S2). The generality of the approach was demonstrated by the polymerization of **1** with 1,10‐decanediol under comparable conditions and yielded a polymeric material that displayed *M*_w_=8.1 kDa (Supporting Information, Figures S3 and S4). Thus POE(II) type materials were accessible without requiring synthesis and isolation of the DETSOU intermediate.

To showcase extension of this method to a wider range of POE structures, and to take advantage of the relatively low molecular weights, we sought to extend our studies to the polymerization of monomers that would yield POEs of type III. At low molecular weights, the inherently flexible polymer backbone equates to POE(III) materials that are typically semisolids with low glass transition temperatures (*T*_g_), which permits convenient mixing with a drug without heating and/or a processing solvent; this property is particularly important for incorporation of sensitive therapeutics.^12^, ^22^ Despite offering numerous advantages, the potential to use such materials is limited by difficulties in polymer synthesis and the reproducibility of the materials; these limitations largely curtailed development of POE(III) from the late 1990s.^11^, ^12^ We postulated that our method could be applicable across all POE platforms and sought to investigate this further.

Preparation of bifunctional A‐B monomers (**7**–**9**), that consist of both the cyclic vinyl acetal and alcohol moieties, was achieved in two simple steps (Scheme 2). Following success with POE(II) from **1**, initially we focused on the retention of the six‐membered ring precursors. Firstly, the triols (**4**–**6**) were synthesized by simple esterification between bis(hydroxymethyl)propionic acid (bis‐MPA) and the corresponding bromoalkanol in *N*,*N*‐dimethylformamide (DMF). This reaction reached maximum conversion at approximately 85 % (with 15 % bis‐MPA starting material). The crude mixture was ring‐closed directly in the subsequent reaction with acrolein to yield the bifunctional monomers **7**–**9**. The ^1^H NMR spectra of the bifunctional monomers (Supporting Information, Figure S5–S7) showed the appearance of vinyl protons (*δ*=5.89–5.26 ppm), which indicated a successful ring closure. Notably, as a consequence of the existence of two chiral centers in the 1,3‐dioxane ring, two diastereomers were observed in each case, which was made evident by the occurrence of two distinct sets of vinyl signals and dioxane ring proton resonance. Polymerization of the bifunctional monomers was undertaken with the optimized conditions for catalyst **3** (Table 1). All materials were characterized by ^1^H NMR spectroscopy (Supporting Information, Figures S9, S11, and S13) and SEC (Supporting Information, Figures S10, S12, and S14), which demonstrated that the polymers displayed *M*_w_ in the range of 8 to 11 kDa. The formation of *endo* and *exo* isomers of the *ortho* ester unit, by addition of the alcohol function above or below the planar ketene acetal function, is indicated by the splitting of the hydrogen atom signals of the 1,3‐dioxane ring in the ^1^H NMR spectra of the polymers. Each polymerization was repeated to demonstrate reproducibility—in stark contrast to typical POE(III) by transesterification, which cannot be prepared reproducibly.^11^, ^12^

**Scheme 2:**
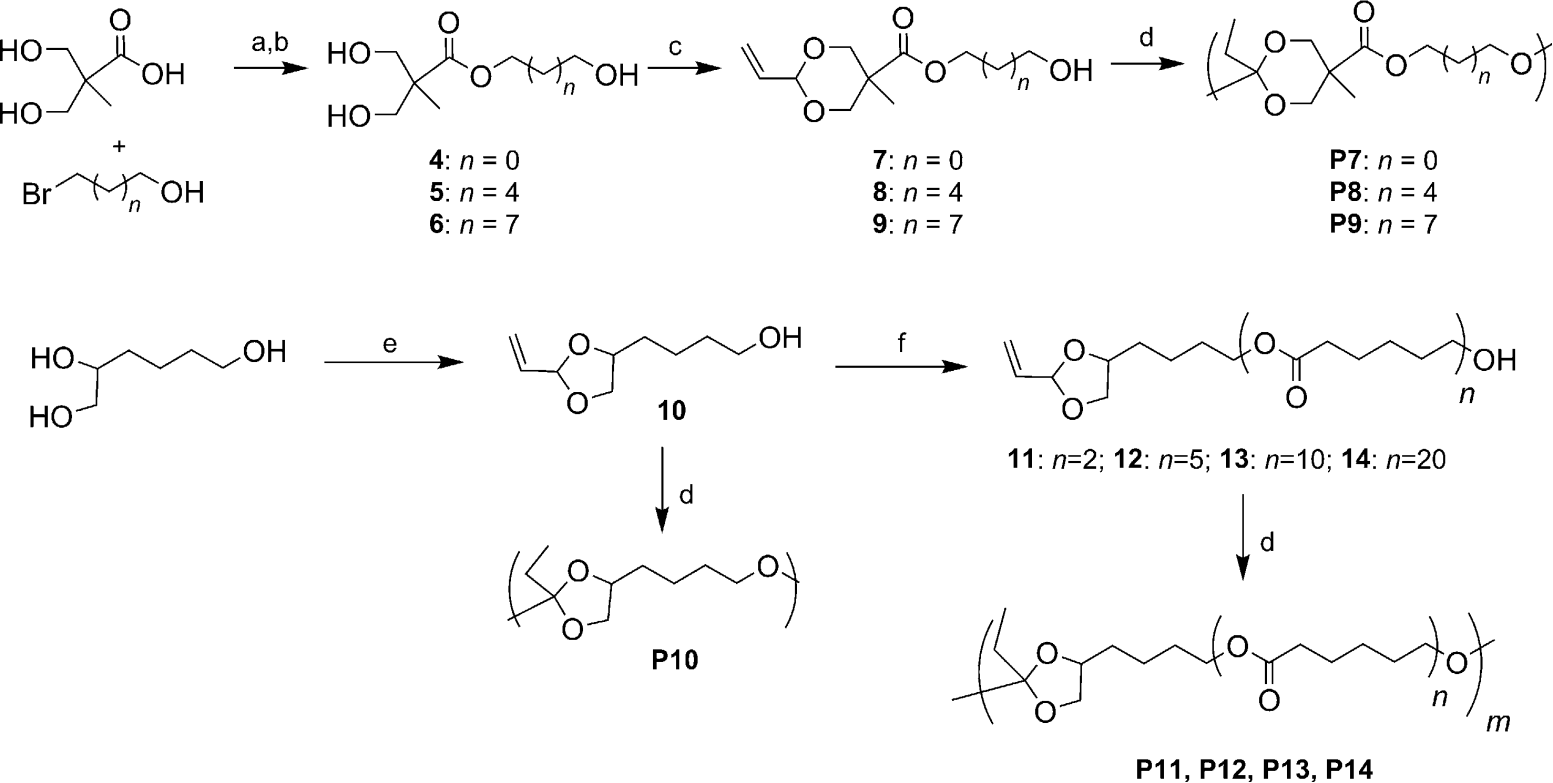
Synthesis and polymerizations of bifunctional monomers **7**–**14**. a) KOH, DMF, 100 °C, 2 h; b) 50 °C, 48 h; c) acrolein, MgSO_4_, *p*‐toluenesulfonic acid (*p*TSA), acetonitrile, 65 °C, 1.5 h; d) **3** (1 mol %), 1,4‐dioxane or toluene, 85 °C, 7–48 h; e) acrolein, *p*TSA, benzene, reflux, 1 h; f) *ϵ*‐caprolactone, Mg(BHT)_2_(THF)_2_, THF.

**Table 1:** Synthesis of POE(III) **P7**–**P14** from bifunctional monomers **7**–**14**.^[a]^

Monomers	*M*_w_ [kDa]^[b]^	*M*_n_ [kDa]^[b]^	*Đ*_M_^[b]^	*T*_g_ [°C]^[c]^	*T*_m_ [°C]^[c]^
**P7**	8.0	5.2	1.53	18	–
**P8**	10.5	7.2	1.47	−23	–
**P9**	11.0	6.8	1.62	−39	–
**P10**	21.2	12.1	1.75	−32	–
**P11**	23.1	10.0	2.32	n.d.	–
**P12**	28.8	15.3	1.88	−57	41
**P13**	43.2	24.6	1.75	−59	51
**P14**	48.8	21.3	2.28	−59	61

[a] Conditions: catalyst **3**, 1,4‐dioxane or toluene, 85 °C, 7–48 h; [b] determined by SEC (CHCl_3_ or THF against polystyrene (PS) standards); [c] determined by DSC analysis.

The ability to apply air‐stable vinyl acetal potentially allows access to a wide range of novel materials that would otherwise be inaccessible by traditional routes. Notably, the instability of ketene acetal precursors to excipient nucleophiles, such as water, increases as the ring size is contracted from six to five.^23^, ^24^ To further demonstrate the utility of this method for the synthesis of new materials, the five‐membered vinyl‐acetal‐containing ring bifunctional monomer (**10**) was isolated directly from the commercially available 1,2,6‐hexanetriol (Figure 2 A).^25^ The subsequent in‐situ‐generated ketene acetal was able to undergo successful step‐growth polymerization (Figures 2 B; Supporting Information, Figures S15 and S16) to yield polymer with *M*_w_ up to 21 kDa.

**Figure 2:**
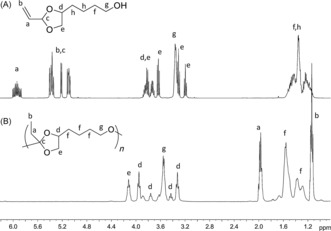
^1^H NMR spectra of A) **10** and B) **P10** in C_6_D_6_ (400 MHz, 25 °C).

The powerful and mild catalytic method presented herein was further applied to access novel materials. Specifically, the preparation of oligo(*ϵ*‐caprolactone)‐based POEs (Scheme 2) was undertaken using the bifunctional monomer **10** as an initiator for the ring‐opening polymerization (ROP) of caprolactone (CL) in the presence of the magnesium catalyst Mg(BHT)_2_(THF)_2_ (BHT=butylated hydroxytoluene; THF=tetrahydrofuran),^26^ which was previously reported for the controlled ROP of lactones.^27^ The new bifunctional macromonomers **11**–**14**, containing oligo(*ϵ*‐caprolactone) from 2 to 20 *ϵ*‐caprolactone units, were obtained in good yield (60–70 %) after purification (Scheme 2 and Figure 3; Supporting Information, Figures S18–S25, Table S1). Macromonomers **13** and **14** were obtained with good control over molecular weight (*M*_n_=1.63 to 3.21 kDa) and dispersity (*Đ*_M_=1.42 to 1.45). As expected, the synthesis of the shorter oligomers, **11** and **12** (2 and 5 equiv. of CL), led to higher dispersity (*Đ*_M_≈2). Monomers **11**–**14** were polymerized in the presence of catalyst **3** to yield POEs **P11**–**P14**. The polymerization proceeded and polymers were produced reproducibly (Supporting Information, Table S2) with *M*_w_ up to about 50 kDa for the higher molecular weight oligomers (**14**), and with lower *M_w_* POEs when the molecular weight of the oligo(*ϵ*‐caprolactone) macromonomer was lower (Supporting Information, Figures S26–S36). POEs **P7**–**P10** are amorphous and do not show any melting peak by differential scanning calorimetry (DSC) analysis. By contrast, the oligo(*ϵ*‐caprolactone)‐based POEs, **P12**–**P14**, showed a melting point (*T*_m_=40 to 61 °C) that results from the presence of the oligo(*ϵ*‐caprolactone) segments in the polymer (Supporting Information, Figures S30, S33, and S36).

**Figure 3:**
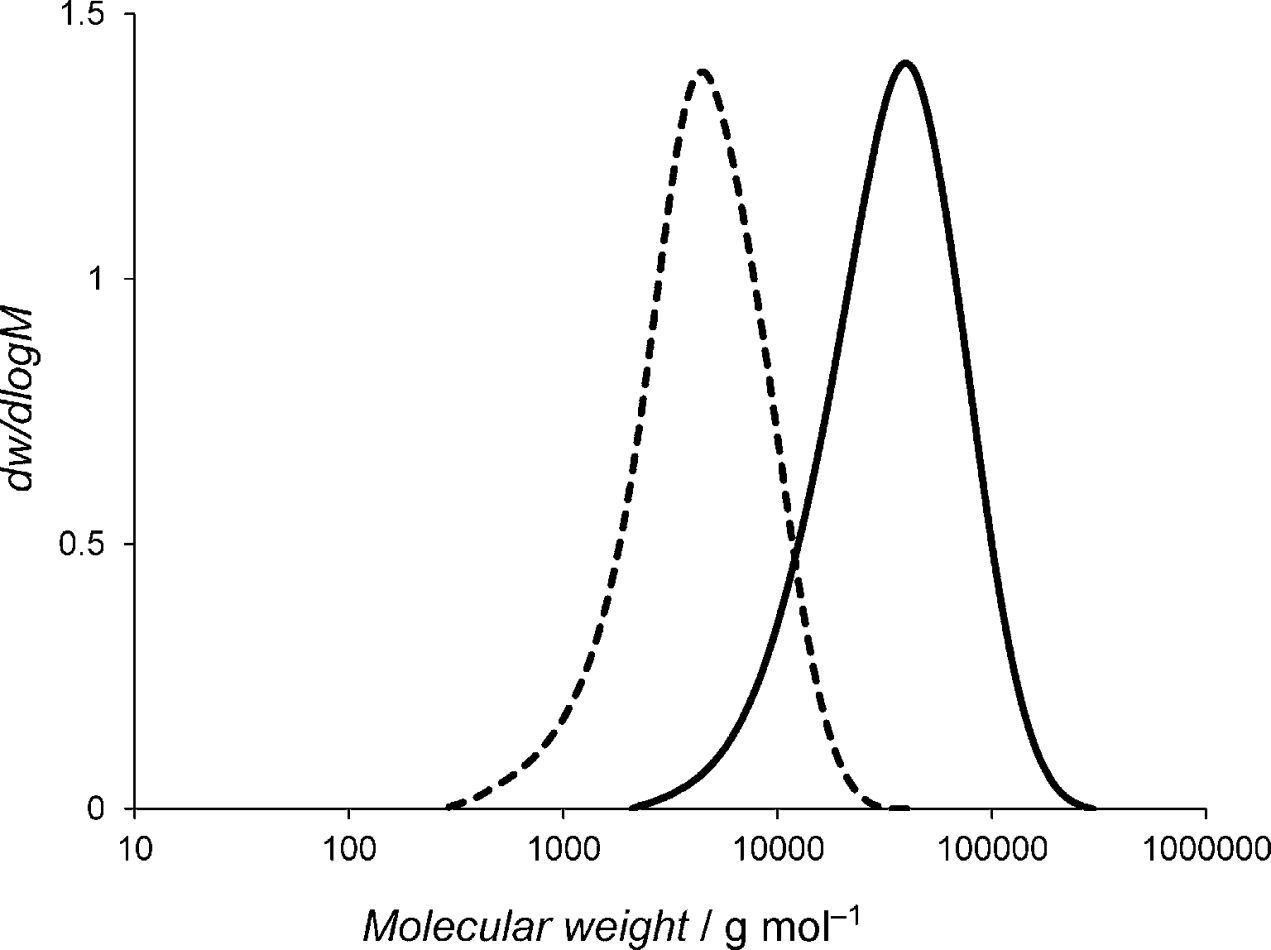
Size‐exclusion chromatograms of **13** (‐ ‐ ‐ ‐) and **P13** (—).

Finally, the mild nature of the process has been further demonstrated by the side‐chain functionalization of a novel degradable aliphatic polycarbonate. The application of *ortho* esters as pH‐responsive side chains^21^, ^28^ has received increased interest over the past few years. While cyclic ketene acetals have been applied for the functionalization of hydroxy‐containing polymers (or derived monomers), the application of such a method to degradable polymers would require protection–deprotection strategies to be employed to overcome the incompatibilities of functional groups with polymerization methods typically required in their synthesis.

To this end, we synthesized a vinyl acetal functional, degradable carbonate polymer, **P15**, by ROP of the corresponding cyclic carbonate monomer, 9‐vinyl‐2,4,8,10‐tetraoxaspiro[5.5]undecan‐3‐one, **15** (Scheme 3). Monomer **15** was prepared in two simple steps starting from pentaerythritol and acrolein to form 2‐vinyl‐1,3‐dioxane‐5,5‐diyldimethanol (Supporting Information, Figure S37), which was subsequently ring‐closed with ethylchloroformate to form **15** in good yield (74 %; Supporting Information, Figure S38). ROP of **15** was achieved using 1,8‐diazabicycloundec‐7‐ene (DBU) with a thiourea cocatalyst,^29^ initiated from benzyl alcohol to realize a polymer with a molecular weight close to that predicted by the monomer:initiator ratio with narrow dispersity. The vinyl‐acetal‐functionalized polycarbonate was subsequently end‐capped with acetic anhydride to form **P15**, with an ester end‐group to prevent undesired cross‐linking reactions in the subsequent steps (Supporting Information, Figure S39).

**Scheme 3:**
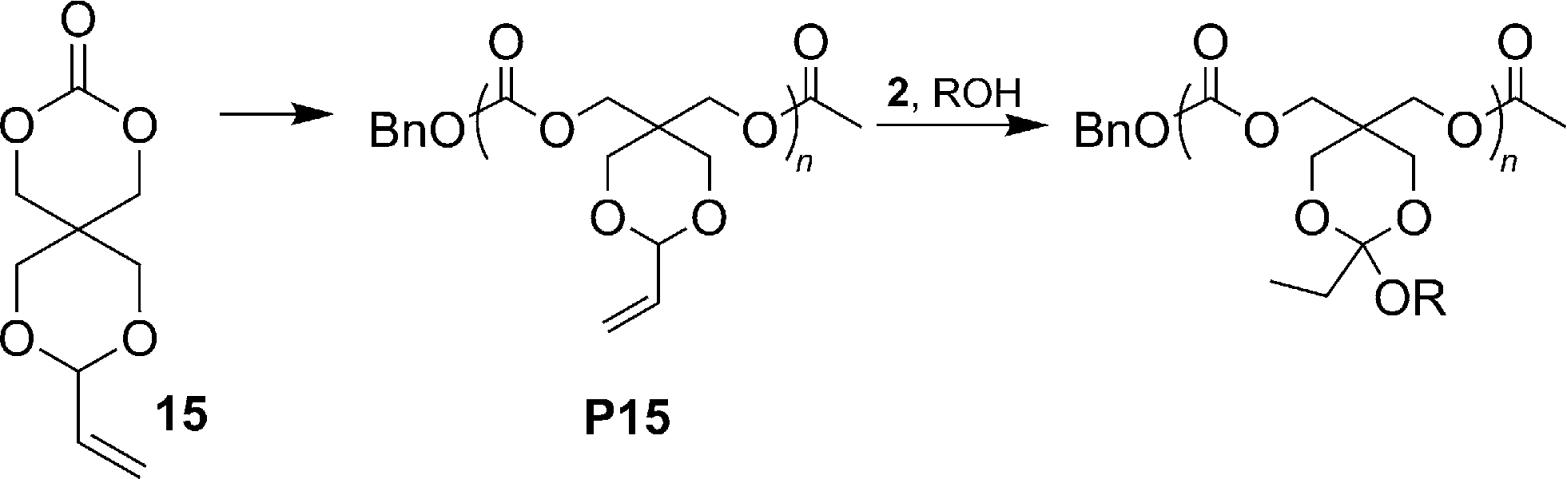
Functionalization of **P15**. Conditions: **2**, 45 °C, 1,4‐dioxane, 12 h.

The vinyl acetal side chains of **P15** were functionalized with 1‐hexanol and benzyl alcohol as model alcohols (Scheme 3). Initial application of catalyst **3** at the higher temperatures required for catalytic operation led to degradation of the polycarbonate backbone. However, application of catalyst **2**, which is highly active at lower temperatures, resulted in polycarbonates with pendant hexyl or benzyl moieties linked through an *ortho* ester. Successful functionalization was evident from the ^1^H NMR spectrum (Supporting Information, Figure S40), showing a distinct upfield shift of the proton resonances from the vinylic (*δ*=5.85–5.25 ppm) to alkyl regions (*δ*=1.72 and 0.92 ppm) and the appearance of the signal (*δ*=3.38 ppm), which is indicative of the attachment of hexyl groups on the side chain of the polycarbonate backbone. SEC analysis (Supporting Information, Figure S41) revealed a single distribution with a comparable dispersity to that of the original **P15**, which indicates that no observable degradation of the polycarbonate backbone occurred. To our knowledge, this is the first report describing generation of an *ortho* ester functional group by addition of alcohol. In turn, this procedure presents a much more versatile method for generating side‐chain substituents that are linked to a polymer backbone by an *ortho* ester.

In summary, the application of RuHCl(PPh_3_)_3_ as catalyst for the synthesis of surface erodible POEs was reported for the first time by a simple and accessible in situ 1,3‐(di)hydride shift of stable (di)vinylacetal moieties in di(bi)functional monomers. In addition, application to bifunctional monomer systems enabled the preparation of POEs that would be extremely difficult, or impossible to prepare in a consistent manner by any other method. As with typical POE(III), the semi‐solid nature of **P7**–**P10** means that these materials may be useful as injectable materials for biomedical applications where viscosity can be easily tuned within a wide range of *T*_g_ (−39 to 18 °C) simply by varying the lengths of the alkyl chain in the bifunctional monomers. We have also demonstrated the versatility of this method by functionalizing an aliphatic polycarbonate by formation of *ortho* ester linkages. The facile nature of this synthetic procedure and the stability of the monomers (compared to other synthetic methods) provide a simple synthetic route to further research into these interesting and highly applicable materials. Our work can potentially provide access to hitherto unprecedented surface erodible materials to potentially enhance the efficacy and control of the current drug delivery systems that rely on bulk degrading materials.

*In memory of Jack Everson*

## Conflict of interest

The authors declare no conflict of interest.

## Supporting information

As a service to our authors and readers, this journal provides supporting information supplied by the authors. Such materials are peer reviewed and may be re‐organized for online delivery, but are not copy‐edited or typeset. Technical support issues arising from supporting information (other than missing files) should be addressed to the authors.

Supplementary
