# Supplementary material for: Unlocking the Potential of Poly(Ortho Ester)s: A General Catalytic Approach to the Synthesis of Surface‐Erodible Materials
Source: Angew Chem Int Ed Engl. 2017 Dec 4;56(52):16664–8. doi: 10.1002/anie.201709934 (PMC5814846; doi:10.1002/anie.201709934)
Supplement: Supplementary file 1 — Supplementary [file ANIE-56-16664-s001.pdf]

Angewandte Chemie
Eine Zeitschrift der Gesellschaft Deutscher Chemiker

Supporting Information

Unlocking the Potential of Poly(*Ortho* Ester)s: A General Catalytic Approach to the Synthesis of Surface-Erodible Materials

*Mathieu J.-L. Tschan+, Nga Sze Ieong+, Richard Todd, Jack Everson†, and Andrew P. Dove**

anie_201709934_sm_miscellaneous_information.pdf

Table of Contents

1. **Model Reaction Studies**……………………………………………………………………………………3
2. **Experimental Section**………………………………………………………………………………………4
   a. General methods……………………………………………………………………………………………4
   b. Synthesis of 5,5-dimethyl-2-vinyl-1,3-dioxane……………………………………………………5
   c. Synthesis of difunctional monomer **1**……………………………………………………………6
   d. Step-growth polymerizations of difunctional monomer **1** with diols………………………6
   e. Bifunctional monomer synthesis **7**-**10** ……………………………………………………………9
      (i) Synthesis of **7**……………………………………………………………………………………9
      (ii) Synthesis of **8**…………………………………………………………………………………11
      (iii) Synthesis of **9**………………………………………………………………………………13
      (iv) Synthesis of **10**………………………………………………………………………………15
   f. Step-growth polymerisations of bifunctional monomers **7 -10….**……………………………16
      (i) Characterization of **P7**……………………………………………………………………16
      (ii) Characterization of **P8**……………………………………………………………………18
      (iii) Characterization of **P9**…………………………………………………………………19
      (iv) Characterization of **P10**…………………………………………………………………20
   g. Preparation of the bifunctional monomers **11**-**14**...22
      (i) General procedure ……………………………………………………………………………22
      (ii) Characterization of **11**……………………………………………………………………23
      (iii) Characterization of **12**…………………………………………………………………24
      (iv) Characterization of **13**…………………………………………………………………26
      (v) Characterization of **14**……………………………………………………………………27
      (vi) Table S1………………………………………………………………………………………29
   h. Step-growth polymerisations of monomers **11**-**14**..29
      (i) General procedure……………………………………………………………………………29
      (ii) Characterization of **P11**…………………………………………………………………29
      (iii) Characterization of **P12**…………………………………………………………………31
      (iv) Characterization of **P13**…………………………………………………………………33

(v) Characterization of **P14**……35
(vi) Table S2……37
i. Synthesis of orthoester-functionalized poly(carbonate)……37
(i) Synthesis of (2-vinyl-1,3-dioxane-5,5-diyl)dimethanol……37
(ii) Synthesis of 9-vinyl-2,4,8,10-tetraoxaspiro[5.5]undecan-3-one (**VDC**)……38
(iii) Synthesis of end-capped **PVDC P15.**……40
(iv) Functionalization of **PVDC** with alcohols……41
3. References……42

1. Model Reaction Studies

Reaction of 5,5-dimethyl-2-vinyl-1,3-dioxane with 1,6-hexanediol catalyzed by complex 2 (or 3) - formation of diorthoester

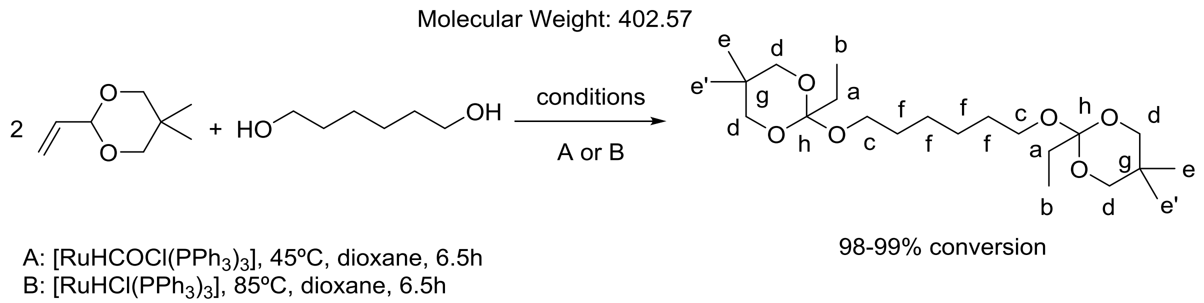

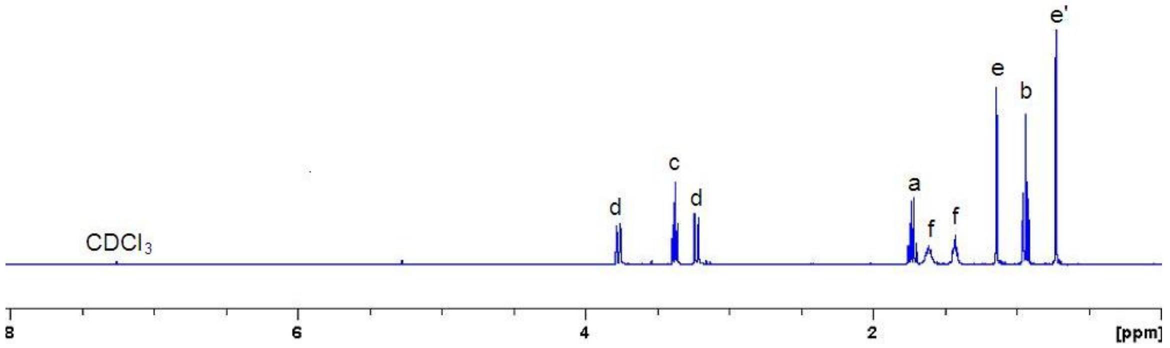

In the glovebox, 5,5-dimethyl-2-vinyl-1,3-dioxane (40 mg, 0.24 mmol) and 1,6-hexanediol (17 mg, 14 mmol) was dissolved in 1,4-dioxane (0.6 mL). The required amount of ruthenium catalyst (**2** or **3**, 2.2 mg, 0.0023 mmol) was loaded on the tip of a glass pipette. The catalyst from the pipette was then washed down a schlenk tube equipped with a Youngs' tap with the above solution of diol and vinylacetal. The schlenk tube was subsequently capped, taken out of the glovebox and then heated in a constant temperature bath (45 °C for catalyst **2** and 85 °C for catalyst **3**) until maximum conversion has been reached (99.7 %, 6.5 h). The crude mixture was then dried under high vacuum before analysis by ^{1}H NMR spectroscopy.

^{1}H NMR (400 MHz, $CDCl_3$, 298 K): δ = 3.77 (d, $^{2}J_{H,H}$ = 10.5 Hz, 4H, H_d), 3.38 (t, $^{3}J_{H,H}$ = 6.6 Hz, 4H, H_c), 3.23 (d, $^{2}J_{H,H}$ = 10.5 Hz, 4H, H_d), 1.71 (q, $^{3}J_{H,H}$ = 7.5 Hz, 4H, H_a), 1.67-1.36 (br m, 8H, H_f), 1.14 (s, 6H, H_e), 0.94 (t, $^{3}J_{H,H}$ = 7.5 Hz, 6H, H_b), 0.73 (s, 6H, $H_{e'}$). $^{13}C\{^{1}H\}$ (100 MHz, $CDCl_3$, 298 K): δ = 112.0 (C_h), 69.9 (C_d), 62.2 (C_c), 29.9 (C_f), 29.2 (C_g), 28.6 (C_a), 26.4 (C_f), 22.8 (C_e), 22.2 ($C_{e'}$), 7.5 (C_b). MS (+ESI): m/z: 425.4 [M^{+} + Na].

2. Experimental Section

a. <u>General Methods</u>

1,8-Diazabicyclo[5.4.0]undec-7-ene (DBU) was dried over CaH_2, distilled and stored under inert atmosphere. Benzyl alcohol was dried and stored over 3 A molecular sieves. 1-(3,5-bis(trifluoromethyl)phenyl)-3-cyclohexylthiourea (TU) was synthesized as reported[1] and dried over CaH_2 in dry THF. [RuHCOCl(PPh_3)$_3$] was synthesized according to the literature procedures and dried over P_2O_5 before use.[2] [RuHCl(PPh_3)$_3$].toluene was purchased from STREM chemicals. Mg(BHT)$_2$(THF)$_2$ was prepared according the literature procedure.[3] $CDCl_3$ was dried over 3 Å molecular sieves, distilled and degassed before use. 1,4-dioxane was refluxed for 3 h over CaH_2 and then further dried over Na/benzophenone ketyl and degassed prior to use. CH_2Cl_2 and THF were purified over Innovative Technology SPS alumina solvent columns and degassed before use. 1,6-hexanediol and 1,12-decanediol were dried over Na at 70 °C and 85 °C respectively for 24 h before being sublimed. 1-hexanol was distilled over Na prior to use. All other solvents and chemicals were obtained from Sigma-Aldrich, Strem or Fischer Scientific and used as received.

Ring-opening polymerizations were performed under inert atmosphere in a glovebox. ^{1}H and ^{13}C{^{1}H} NMR spectra were recorded on a Bruker DPX-400 spectrometer at 298 K. Chemical shifts are reported as δ in parts per million (ppm) and referenced to the chemical shift of the residual solvent resonances (C_6H_6: ^{1}H δ = 7.16 ppm; ^{13}C{^{1}H} δ = 128.06 ppm, $CHCl_3$: ^{1}H δ = 7.26 ppm; ^{13}C{^{1}H} δ = 77.16 ppm, $(CH_3)_2SO$ ^{1}H δ = 2.50 ppm; ^{13}C{^{1}H} δ = 39.52 ppm, MeOH ^{1}H δ = 3.31 ppm; ^{13}C{^{1}H} δ = 49.00 ppm, H_2O: ^{1}H δ = 4.79 ppm). Mass spectra were recorded on a Bruker HCT+ ESI spectrometer. Elemental analysis was performed in duplicate by Warwick Analytical Services. Size exclusion chromatography (SEC) at 30 °C was conducted on a system composed of a Varian 390-LC-Multi detector suite fitted with differential refractive index, light scattering, and viscometer detectors equipped with a guard column (Varian Polymer Laboratories PLGel 5 µM, 50 × 7.5 mm) and two mixed D columns (Varian Polymer Laboratories PLGel 5 µM, 300 × 7.5 mm). The mobile phase was $CHCl_3$ or THF with 2 % TEA at a flow rate of 1.0 mL min^{-1}. SEC samples were calibrated against Varian Polymer Laboratories Easi-Vials linear poly(styrene) (PS) standards (162-2.4 × 10^5 g mol^{-1}) using Cirrus v3.3 software. DSC measurements were performed on a Mettler Toledo, HP DSC827 with analysis performed

using Mettler Toledo STARe software v9.20. The samples were run at a heating rate of 10 °C min^{-1}, and the glass transition temperatures (T_gs) were taken as the midpoint of the inflection tangent. Melting points were determined on an OptiMelt Automated Melting Point System (SRS).

b. <u>Synthesis of 5,5-dimethyl-2-vinyl-1,3-dioxane</u>

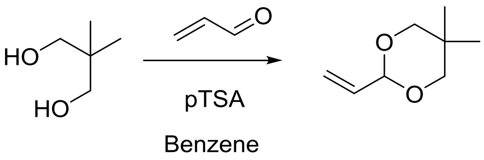

To a pre-dried three-neck round bottom flask equipped with a reflux condenser was added 2,2-dimethyl-1,3-propanediol (10 g, 96 mmol), hydroquinone (0.12 g, 0.96 mmol), and *p*-toluenesulfonic acid (*p*TSA) (0.88 g, 4.6 mmol) under nitrogen. Benzene (60 mL) was then added and the reaction mixture was heated to 75 °C after which acrolein (7.1 mL, 110 mmol) was added dropwise via a syringe. The reaction was then stirred for 1.5 h after which it was allowed to cool to room temperature before the benzene and excess acrolein were removed under reduced pressure to yield a viscous yellow oil. The crude product was then distilled under static vacuum to give a colourless oil. This was then subject to column chromatography (silica gel, eluent CH_2Cl_2) to give 5,5-dimethyl-2-vinyl-1,3-dioxane (6.5 g, 48 %, TLC: R_f 0.55 inCH_2Cl_2). The pure product was then dried over CaH_2 overnight prior to distillation under static vacuum at 35 °C before being stored in the freezer at -30 °C.

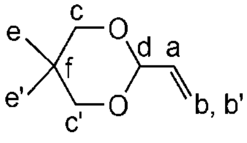

^{1}H NMR (400 MHz, $CDCl_3$, 298 K): δ= 5.92-5.82 (m, 1H, H_a), 5.48-5.27 (m, 2H, $H_{b,b'}$), 4.83 (d, $^3J_{H,H}$ = 4.0 Hz, 1H, H_d), 3.65-3.45 (m, 4H, $H_{c,c'}$), 1.21, 0.73 (s, 6h, $H_{e,e'}$). ^{13}C{^{1}H} (100 MHz, $CDCl_3$, 298 K): δ= 135.2 (C_a), 119.2 ($C_{b,b'}$), 101.2 (C_d), 77.4 (C_c), 30.3 (C_f), 23.3 ($C_{e,e'}$), 22.0 ($C_{e,e'}$). Characterizations are analogous to those reported previously.[3]

c. Synthesis of difunctional monomer 1

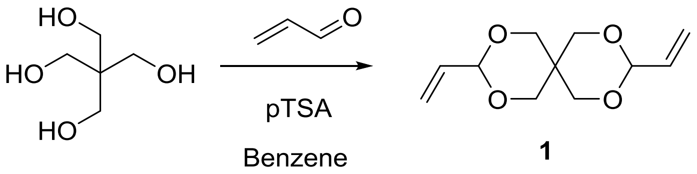

Compound **1** was synthesized using a similar procedure to 5,5-dimethyl-2-vinyl-1,3-dioxane using pentaerythritol (20 g, 0.15 mol), acrolein (24.7 g, 0.44 mol), *p*TSA (1.4 g, 7.4 mmol) in benzene (200 mL). The crude product was purified by column chromatography (silica gel, eluent CH_2Cl_2/Et_2O (v/v) 100:1 to 100:4 gradient) to give **1** as a white solid (17.8 g, 56 %, TLC: R_f 0.46 $EtOAc/CH_2Cl_2$ (v/v) 1:9). The difunctional monomer was then dried over CaH_2 at 70 °C for 24 h and then sublimed twice prior to being stored in the glovebox at -30 °C.

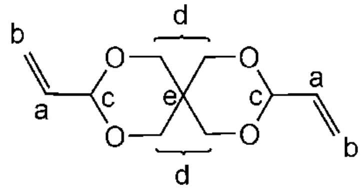

^{1}H NMR (400 MHz, $CDCl_3$, 298 K): δ= 5.86 (m, 2H, H_a), 5.50-5.31 (m, 4H, H_b), 4.88 (d, $^3J_{H,H}$ = 4.5 Hz, 2H, H_c), 4.64, 3.65, 3.47, 3.42 (m, 8H, H_d). $^{13}C\{^1H\}$ (100 MHz, $CDCl_3$, 298 K): δ= 134.5 (C_a), 119.5 (C_b), 101.7 (C_c), 70.6, 70.2 (C_d), 32.5 (C_e). Characterizations are analogous to those reported previously.[4]

d. Step-growth polymerizations of difunctional monomer 1 with diols

The polymerization of divinylacetal and 1,6-hexanediol was given as an example. In the glovebox, divinylacetal (0.4995 g, 2.33 mmol) and 1,6-hexanediol (0.2758 g, 2.33 mmol) was dissolved in 1,4-dioxane (2 mL). Catalyst **3** (5 mg, 0.0049 mmol, 2 mol %), loaded onto the tip of pipette, was washed down into a Schlenk tube with the monomer solution. The reaction vessel was then capped, taken out of the glovebox and heated in an oil bath at 85 °C for the required time (0.5 h – 70 h). After that, the reaction was quenched in an ice

bath, concentrated under reduced pressure and precipitated into methanol. Finally the recovered polymer was dried prior to analysis by NMR spectroscopy and GPC.

*Characterization of POE from step-growth polymerization of **1** and 1,6-hexanediol*

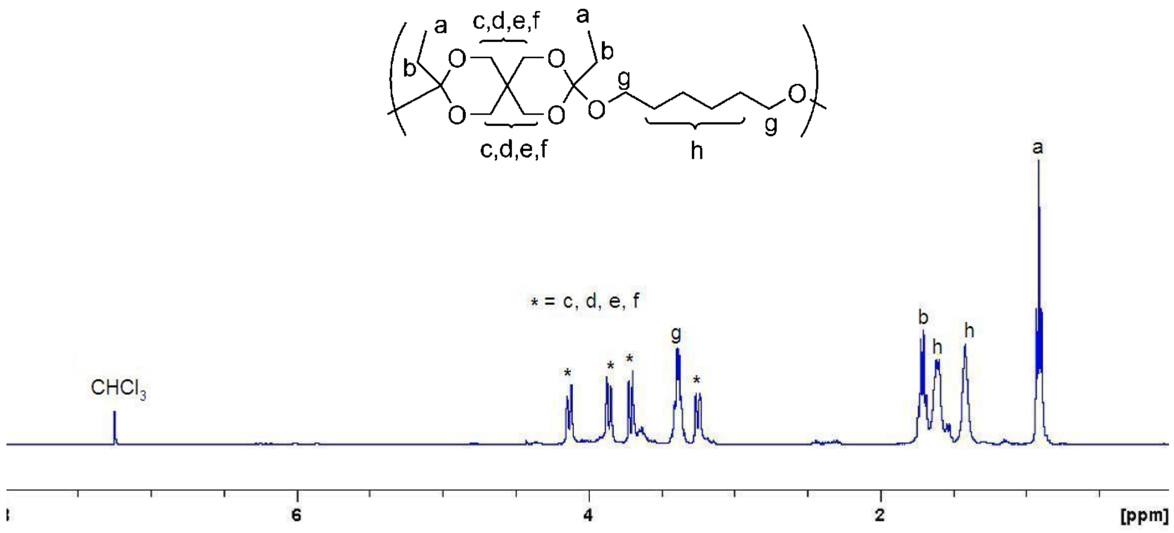

Figure S1. ^{1}H NMR spectrum *POE from step-growth polymerization of **1** and 1,6-hexanediol* in $CDCl_3$ (400 MHz, 298 K).

Yield 70-75 %. ^{1}H NMR (400 MHz, $CDCl_3$, 298 K): δ = 4.14 (m, 2H, $H_{c,d,e,f}$), 3.86 (br m, 2H, $H_{c,d,e,f}$), 3.72 (m, 2H, $H_{c,d,e,f}$), 3.39 (m, 4H, H_g), 3.25 (m, 2H, $H_{c,d,e,f}$), 1.71 (br q, $^3J_{H,H}$ = 7.4 Hz, 4H, H_b), 1.61, 1.44 (br m, 8H, H_h), 0.92 (br t, $^3J_{H,H}$ = 6.9 Hz, 6H, H_a). GPC ($CHCl_3$, PS standards) M_w = 9.5 kDa, $Đ_M$ = 1.59. Characterizations are comparable to those reported previously.[5]

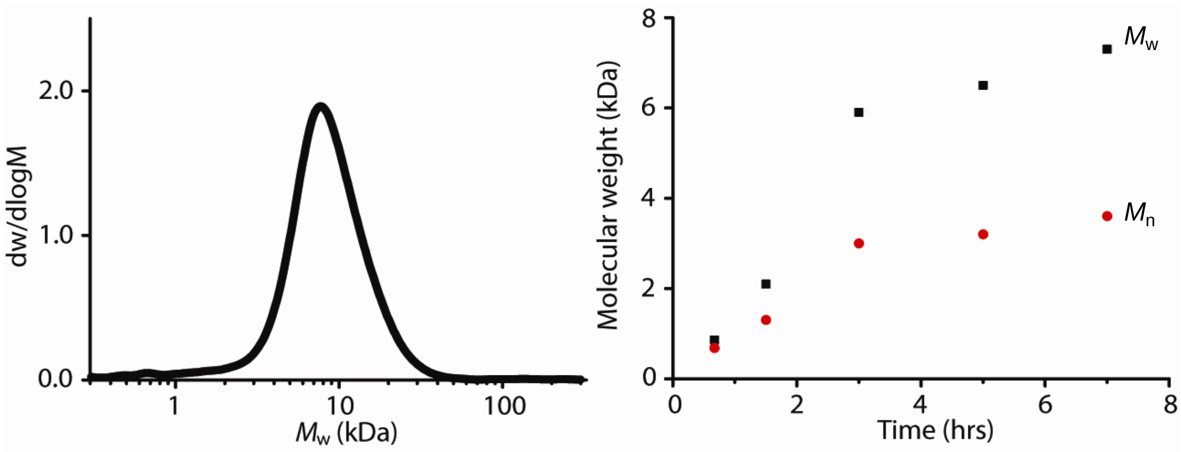

Figure S2. (a) SEC chromatogram ($CHCl_3$ eluent, PS standards), (b) molecular weight *versus* time plot of POE from step-growth polymerization of **1** and 1,6-hexanediol (.

*Characterization of POE from step-growth polymerization of **1** and 1,10-decanediol*

Yield 77-80 %. ^{1}H NMR (400 MHz, C_6D_6, 298 K): δ = 4.59 (m, 2H, $H_{c,d,e,f}$), 4.15 (br m, 2H, $H_{c,d,e,f}$), 3.84 (m, 2H, $H_{c,d,e,f}$), 3.50 (m, 4H, H_g), 3.27 (m, 2H, $H_{c,d,e,f}$), 1.96 (br q, $^3J_{H,H}$ = 6.9 Hz, 4H, H_b), 1.73, 1.53, 1.44, 1,39 (br m, 16H, H_h), 1.21 (br t, $^3J_{H,H}$ = 7.9 Hz, 6H, H_a). ^{13}C{^{1}H} (100 MHz, C_6D_6, 298 K): δ = 112.7 (C_i), 63.3 (C_j), 62.5 ($C_{c,d,e,f}$), 62.1 (C_g), 30.3, 30.0, 26.9, 20.7 (C_h), 28.8 (C_b), 7.7 (C_a). GPC ($CHCl_3$, PS standards) M_w = 8.0 kDa, $Đ_M$ = 1.36.

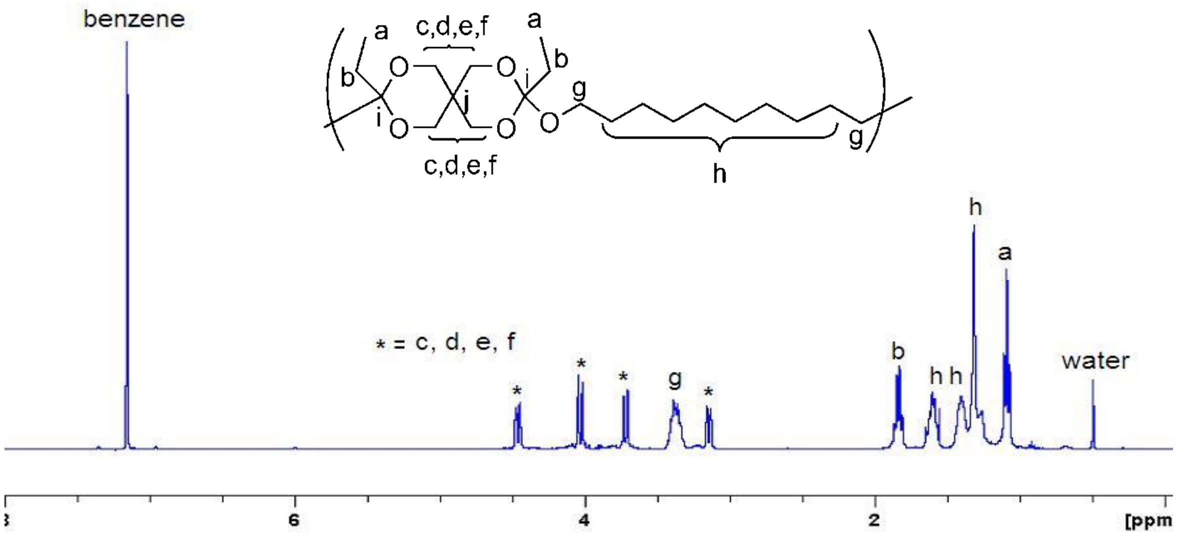

Figure S3. ^{1}H NMR spectrum of **P6** in C_6D_6 (400 MHz, 298 K).

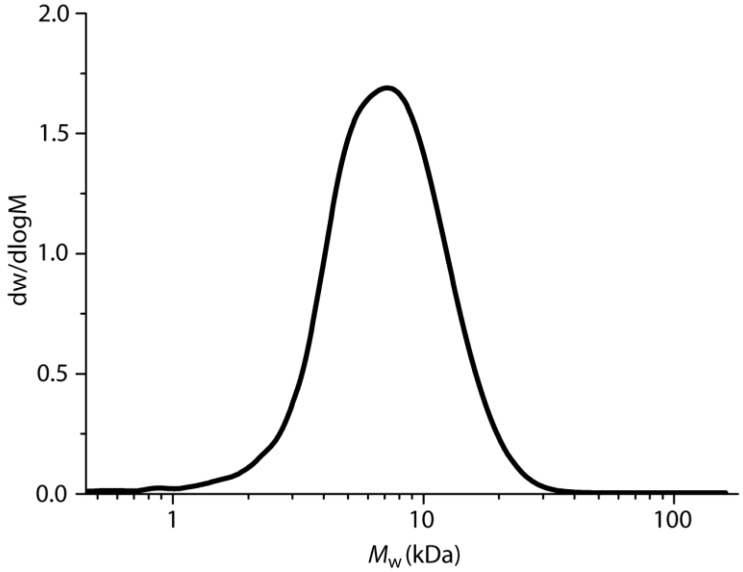

Figure S4. SEC chromatogram ($CHCl_3$ eluent, PS standards) of POE from step-growth polymerization of **1** and 1,10-decanediol.

e. Bifunctional monomer synthesis

(i) Synthesis of 7

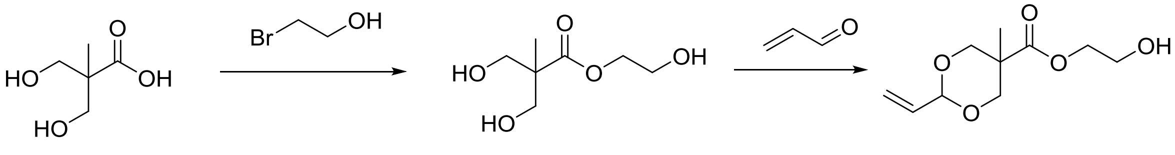

*Synthesis of intermediate **4**:* In a round bottom flask, bis-MPA (90 g, 0.67 mol) and KOH (43.3 g, 0.77 mol) were heated at 100 °C in DMF (500 mL) for 2 h. The reaction temperature was then reduced to 45 °C after which 2-bromo-ethanol (92.7 g, 0.74 mol) was added dropwise. The mixture was then heated for a further 48 h. The DMF was removed under reduced pressure to give a white semi-solid which was then extracted with ethanol (2 × 250 mL). The precipitated KBr was filtered off and the solvent removed from the resulting crude mixture on a rotary evaporator to yield a translucent semi-solid.

Analysis by ^{1}H NMR spectroscopy indicated the formation of the desired triol in 85 % conversion (quantitative yield (85 % (102 g of triol product) + 15 % (13.5 g of bis-MPA)). The product was used in the next step without further purification.

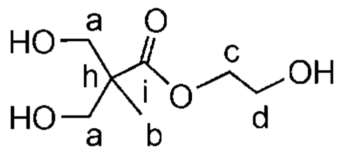

*Characterization of triol **4***: ^{1}H NMR (400 MHz, MeOD, 298 K): δ= 4.19-4.17 (m, 2H, H_c), 3.76-3.73 (n, 2H, H_d), 3.73-3.62 (m, 4H, H_a), 1.17 (s, 3H, H_b), OH not observed. $^{13}C\{^{1}H\}$ (100 MHz, D_2O, 298 K): δ= 176.9 (C_i), 66.4 (C_c), 64.6 (C_a), 59.6 (C_d), 50.3 (C_h), 16.0 (C_b). MS (+ESI): *m/z*: 201 [M^+ + Na], 217 [M^+ + K].

*Synthesis of **7**:* In a 3-neck round bottom flask equipped with a condenser was added the triol (23 g, 0.13 mol), acetonitrile (150 mL) and $MgSO_4$ (*ca*. 7 g) under nitrogen. The suspension was then stirred at 65 °C for 15 min after which acrolein (9.5 mL, 7.97 g, 0.14 mol) was added dropwise via a dropping funnel over the course of 15 min. The resulting reaction mixture was stirred for a further 2 h and then cooled to room temperature. The salt was then filtered by gravity and the acetonitrile removed on a rotary evaporator. The crude product was extracted with ethylacetate (50 mL) and subsequently washed with saturated $NaHCO_3$ (50 mL). The aqueous layer was then extracted with ethylacetate (2 × 50 mL) and the combined organic layers dried with $MgSO_4$ to yield an orange oil (crude yield 14 g, 50 %) that was then further purified by column chromatography (silica gel, eluent CH_2Cl_2/EtOAc (v/v) 10:1 to 1:1 gradient) to give **7** (8.0 g, 29 %, R_f 0.40 minor isomer; 0.30 major isomer EtOAc/CH_2Cl_2 (v/v) 1:9). The product was then taken up in anhydrous toluene, dried over CaH_2 overnight prior to distillation (2 x 10^{-2} mbar, 90 °C) to afford a clear colourless liquid before being stored in the glove box freezer at -30 °C. It should be noted that the ^{1}H NMR spectroscopic analysis indicated the presence of two diastereomers in the ratio of 7:93.

^{1}H NMR (400 MHz, $CDCl_3$, 298 K): δ= 5.89-5.84 (m, 1H, H_a (minor isomer)), 5.83-5.75 (m, 1H, H_a (major isomer)), 5.48-5.30 (m, 2h, $H_{b,b'}$, minor isomer), 5.45-5.26 (m, 2h, $H_{b,b'}$, major

isomer), 4.91 (d, $^{3}J_{H,H}$ = 4.7 Hz, 1H, H_c, major isomer), 4.85 (d, $^{3}J_{H,H}$ = 4.5 Hz, 1H, H_c, minor isomer), 4.85-3.48 (m, 4H, H_d, major isomer), 4.34-4.31 (m, 2H, H_g, major isomer), 4.22-4.19 (m, 2H, H_g, minor isomer), 3.96 (s, 4H, H_d, minor isomer), 3.79-3.77 (m, 2H, H_f, major isomer), 1.51 (s, 3H, H_e, minor isomer), 0.99 (s, 3H, H_e, major isomer). $^{13}C\{^{1}H\}$ (100 MHz, $CDCl_3$, 298 K): δ= 174.8 (C_i, major isomer), 134.5 (C_a, major isomer), 119.7 (C_b, major isomer), 101.2 (C_c, major isomer), 73.6 (C_d, major isomer), 72.1 (C_d, minor isomer), 66.8 (C_g, major isomer), 61.1 (C_f, major isomer), 43.1 (C_h, major isomer), 19.5 (C_e, minor isomer), 17.5 (C_e, major isomer). Anal Calcd for $C_{10}H_{16}O_5$: C 55.55; H 7.46 %. Found: C 55.22; H 7.48 %. MS (+ESI): *m/z*: 239 [M^+ + Na], 216 [M^+].

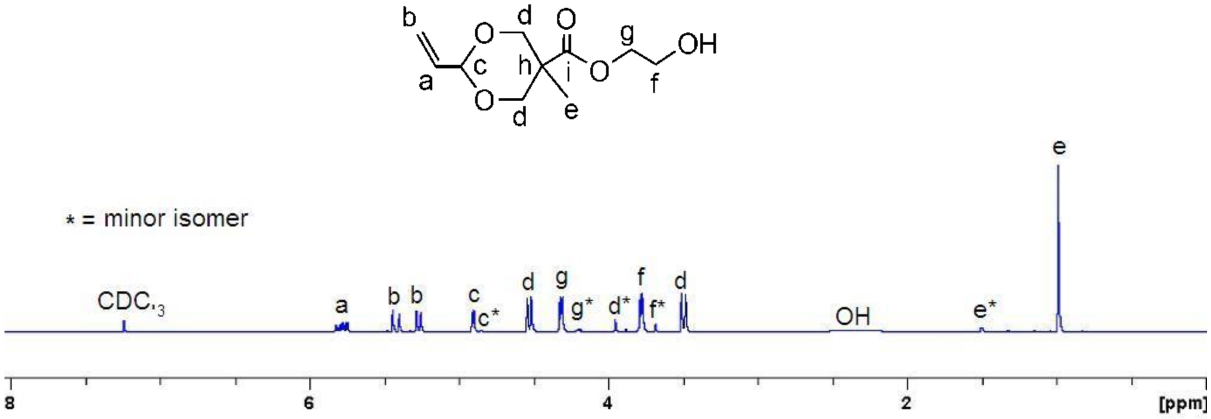

Figure S5. ^{1}H NMR spectrum of bifunctional monomer **7** in $CDCl_3$ (400 MHz, 298 K).

(ii) Synthesis of 8

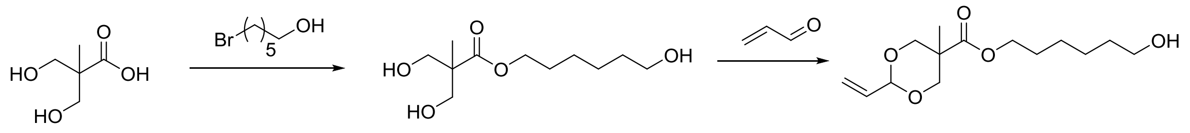

5 **8**

*Synthesis of intermediate **5**:* In a round bottom flask was added bis-MPA (8.1 g, 61 mmol), KOH (3.4 g, 61 mmol) and DMF (36 mL) and the mixture was then heated at 100 °C for 3 h. The reaction was then cooled to 55 °C after which 6-bromo-1-hexanol (10 g, 55 mmol) was added dropwise over 1 h. The resulting mixture was stirred for a further 2 days. Analysis by ^{1}H NMR spectroscopy indicated that the reaction had proceeded to *ca*. 70% conversion

to the triol (30% unreacted bis-MPA). The precipitated salt was then filtered and solvent removed *in vacuo*. The crude mixture was taken up in ethyl acetate (50 mL) and washed with saturated $NaHCO_3$ (50 mL). The aqueous layer was washed with ethyl acetate (3 × 50 mL) and the combined organic layers were dried with $MgSO_4$. Removal of solvent under reduced pressure gave **5** as a light-yellow oil that was used without further purification (7.4 g, 82% (based on 70% conversion in 1st step)).

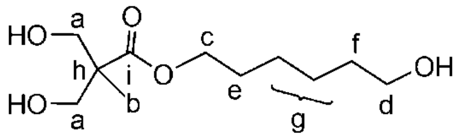

^{1}H NMR (400 MHz, D_2O, 298 K): δ= 4.17 (t, $^3J_{H,H}$ = 6.2 Hz, 2H, H_c), 3.78-3.64 (m, 4H, H_a), 3.61 (t, $^3J_{H,H}$ = 6.7 Hz 2h, H_d), 1.74-1.64 (br m, 2h, H_e), 1.62-1.51 (br m, *2*H, H_f), 1.50-1.31 (br m, 4H, H_g), 1.18 (s, 3H, H_b). $^{13}C\{^1H\}$ (100 MHz, D_2O, 298 K): δ= 177.3 (C_i), 65.7 (C_c), 64.6 (C_a), 61.7 (C_d), 50.3 (C_h), 31.1 (C_f), 27.6 (C_e), 24.9 (C_g), 24.6 (C_g), 16.1 (C_b). MS (+ESI): *m/z*: 257 [M^+ + Na].

Synthesis of **8***:* Compound **8** was synthesized using a similar procedure to **7** using triol **5** (7.4 g, 32 mmol), acrolein (2.23 g, 35 mmol), H_2SO_4 on silica (0.18 g) $MgSO_4$ (ca. 6 g) in acetonitrile (36 mL). The crude product was purified by column chromatography (silica gel, eluent CH_2Cl_2/Et_2O (v/v) 4:1) to give **8** (1.7 g, 20% overall yield, R_f 0.50 and 0.36 two isomers 100% Et_2O). It should be noted that the ^{1}H NMR spectroscopic analysis indicated the presence of two diastereomers in the ratio of 35:65. The bifunctional monomer was then dried over P_2O_5 prior to being stored in the glovebox at -30 °C.

^{1}H NMR (400 MHz, $CDCl_3$, 298 K): δ= 5.90-5.77 (m, 1H, H_a (both isomers)), 5.49-5.25 (m, 2h, H_b (both isomers)), 4.89 (d, $^3J_{H,H}$ = 5.6 Hz, 1H, H_c, major isomer), 4.85 (d, $^3J_{H,H}$ = 5.6 Hz, 1H, H_c, minor isomer), 4.55-3.46 (m, 4H, H_d, major isomer), 4.18 (t, $^3J_{H,H}$ = 6.7 Hz, 2H, H_g, major isomer), 4.08 (t, $^3J_{H,H}$ = 6.7 Hz, 2H, H_g, minor isomer), 4.22-4.19 (m, 2H, H_g, minor isomer), 3.94 (s, 4H, H_d, minor isomer), 3.62 (br m, 2H, H_f, major isomer), 1.66 (br m, 2H, H_k, both isomers), 1.55 (br m, 2H, H_l, both isomers), 1.49 (s, 3H, H_e, minor isomer), 1.39 (br m, 4H, H_j, both isomers), 0.99 (s, 3H, H_e, major isomer). $^{13}C\{^1H\}$ (100 MHz, $CDCl_3$, 298 K): δ= 174.0 (C_i, both isomers), 134.2 (C_a, both isomers), 119.2 (C_b, both isomers), 101.1 (C_c, major isomer), 100.8 (C_c, minor isomer), 73.2 (C_d, major isomer), 72.1 (C_d, minor isomer),

65.0 (C_g, major isomer), 64.7 (C_g, minor isomer), 62.7 (C_f, major isomer), 42.6 (C_h, major isomer), 40.8 (C_h, minor isomer), 32.6 (C_l, both isomers), 28.5 (C_k, both isomers), 25.5 (C_j, both isomers), 19.3 (C_e, minor isomer), 17.9 (C_e, major isomer). Anal Calcd for $C_{14}H_{24}O_5$: C 61.74; H 8.88 %. Found: C 61.66; H 8.96 %. MS (+ESI): *m*/*z*: 295 [M^+ + Na], 273 [M^++ H].

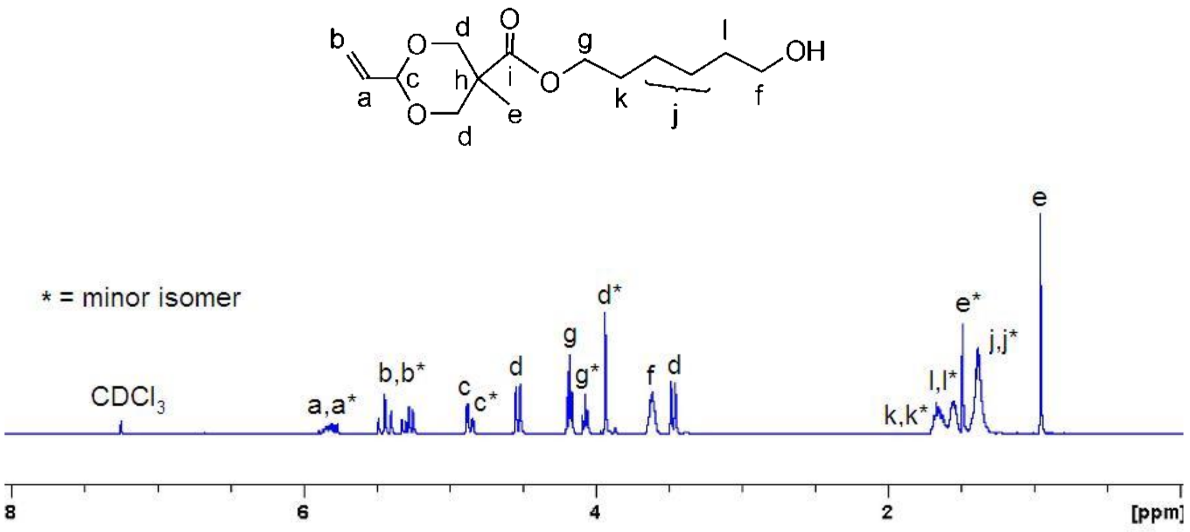

Figure S6. ^{1}H NMR spectrum of bifunctional monomer **8** in $CDCl_3$ (400 MHz, 298 K).

(iii) Synthesis of 9

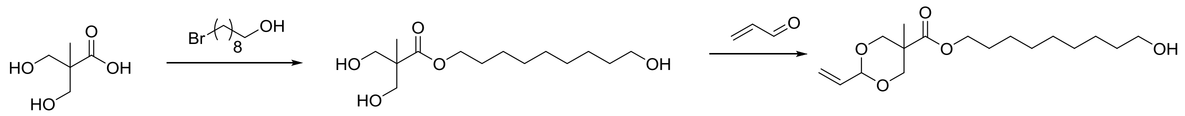

6 **9**

Synthesis of intermediate **6**: In a round bottom flask was added bis-MPA (16.5 g, 0.12 mol) and KOH (6.9 g, 0.12 mol) in DMF (75 mL). The mixture was heated at 100 °C for 3 h after the temperature was reduced to 50 °C. 9-bromo-1-nonanol (25 g, 0.11 mol) dissolved in DMF (ca. 10 mL) was added via a dropping funnel over 1 h. The resulting suspension was then allowed to stir at 50 °C for 3 days. Analysis by ^{1}H NMR spectroscopy indicated *ca*. 70 % conversion to the triol (30 % unreacted bis-MPA). After cooling the mixture to room temperature, the KBr salt was filtered by gravity and the DMF removed *in vacuo* to yield a light orange viscous oil which was subsequently taken up in EtOAc (100 mL) and washed

with saturated $NaHCO_3$ (100 mL). The aqueous layer was further extracted with EtOAc (2 × 50 mL) and the combined organic layer dried with $MgSO_4$. Evaporated of solvent under reduced pressure gave triol **6** (9.6 g, 46% based on 70% conversion) which was directly used in the next step without further purification.

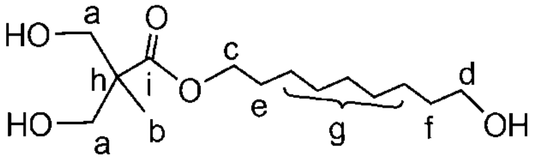

*Characterization of triol **6***: ^{1}H NMR (400 MHz, MeOD, 298 K): δ = 4.10 (t, $^3J_{H,H}$ = 6.3 Hz, 2H, H_c), 3.70-3.61 (m, 4h, H_a), 3.52 (t, $^3J_{H,H}$ = 6.3 Hz, 2H, H_d), 1.69-1.62 (m, 2H, H_e), 1.58-1.50 (m, 2H, H_f), 1.34 (br m, 10H, H_g), 1,16 (s, 3H, H_b), OH not observed. ^{13}C{^{1}H} (100 MHz, D_2O, 298 K): δ = 176.8 (C_i), 65.7 (C_c), 65.6 (C_d), 62.9 (C_a), 51.3 (C_h), 33.5 (C_f), 30.5, 30.4, 30.2, 26.9, 26.8 (C_g), 29.6 (C_e), 17.3 (C_b). MS (+ESI): *m/z*: 295 [M^+ + Na], 273 [M^++ H]

Synthesis of **9**: Compound **9** was synthesized using a similar procedure to **7** using triol **6** (10 g, 36 mmol), acrolein (2.23 g, 40 mmol), H_2SO_4 on silica (0.54 g) $MgSO_4$ (ca. 6 g) in acetonitrile (50 mL). The crude product was purified by column chromatography (silica gel, eluent Et_2O/petroleum ether (v/v) 3:7 to 1:1 gradient) to give **9** as light green viscous oil (1.2 g, 11 %, R_f 0.12, Et_2O/petroleum ether (v/v) 4:6). It should be noted that as the minor isomer (R_f 0.22) co-elute with other unidentified impurities, only the major isomer was isolated and fully characterized for subsequent polymerizations. The bifunctional monomer was then dried over P_2O_5 prior to being stored in the glovebox at -30 °C.

^{1}H NMR (400 MHz, $CDCl_3$, 298 K): δ = 5.86-5.78 (m, 1H, H_a), 5.45-5.26 (m, 2h, H_b), 4.89 (d, $^3J_{H,H}$ = 5.0 Hz, 1H, H_c), 4.55-3.46 (m, 4H, H_d), 4.17 (t, $^3J_{H,H}$ = 6.2 Hz, 2H, H_g), 3.62 (br m, 2H, H_f), 1.68-1.60 (br m, 2H, H_k), 1.59-1.51 (br m, 2H, H_j), 1.29 (br m, 10, H_l), 1.49 (s, 3H, H_e, minor isomer), 0.97 (s, 3H, H_e). ^{13}C{^{1}H} (100 MHz, $CDCl_3$, 298 K): δ = 174.5 (C_i), 134.7 (C_a), 119.5 (C_b), 101.4 (C_c), 73.4 (C_d), 65.4 (C_g), 63.6 (C_f), 42.8 (C_h), 33.1 (C_j), 29.6; 29.5 (C_l), 29.2 (C_k), 25.9 (C_l), 18.2 (C_e). Anal Calcd for $C_{17}H_{30}O_5$: C 64.94; H 9.62 %. Found: C 64.91; H 9.69 %. MS (+ESI): *m/z*: 337 [M^+ + Na], 315 [M^++ H]

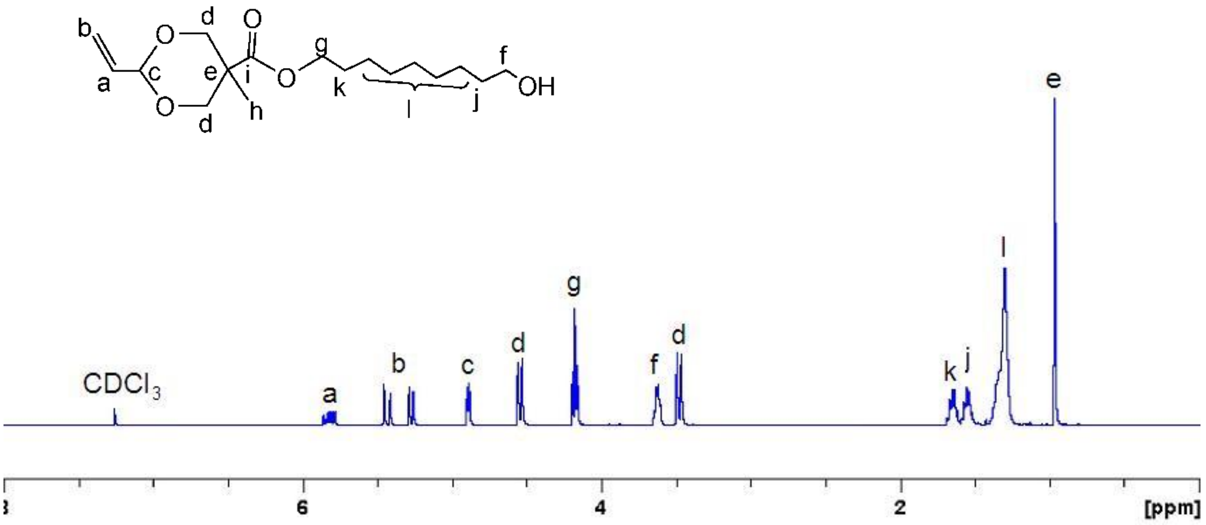

Figure S7. ^{1}H NMR spectrum of bifunctional monomer **9** in $CDCl_3$ (400 MHz, 298 K).

(iv) Synthesis of 10

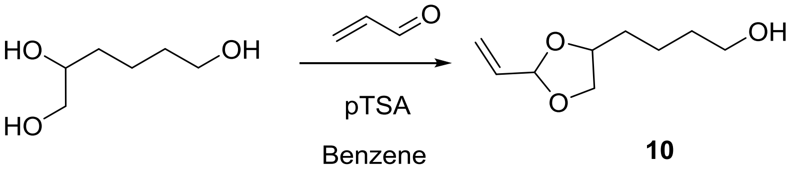

Compound **10** was synthesized following the procedure reported by Smith et al.[6] The crude product was purified by short-path distillation under vacuum (using a Kugelrohr) to give **10** as a colorless oil (yield 60%). The bifunctional monomer was dried over CaH_2 overnight, cryo-distilled under vacuum and stored in the glovebox at -30 °C.

^{1}H NMR (400 MHz, $CDCl_3$, 298 K): δ= 5.90-5.74 (m, 1H, H_a), 5.56-5.30 (m, 2h, H_b), 5.39-5.18 (m, 1H, H_c), 4.16-3.44 (m, 2H, H_e), 4.10 (m, 2H, H_d), 3.61 (t, $^3J_{H,H}$ = 6.8 Hz, 2H, H_g), 1.84-1.17 (br m, 4H, H_h), 1.55 (br m, 2H, H_f). $^{13}C\{^1H\}$ (100 MHz, $CDCl_3$, 298 K): δ= 134.8 (C_a), 120.5, 119.8 (C_b), 104.1, 103.4 (C_c), 77.1, 76.2 (C_d), 70.4, 69.8 (C_e), 62.6 (C_g), 33.2, 32.7,22.2 (C_h), 32.9 (C_f) (note: In $CDCl_3$, only 16 carbons were observed in the ^{13}C NMR spectra of **10** obtained as two isomers). $^{13}C\{^1H\}$ (100 MHz, C_6D_6, 298 K): 136.04, 136.02 (C_a), 119.33, 118.58 (C_b), 104.34, 103.57 (C_c), 77.19, 76.22 (C_d), 70.36, 69.81 (C_e), 62.36,

62.34 (C_g), 33.41, 33.18, 32.91, 32.89, 22.50, 22.48 (C_h, C_f). Anal Calcd for $C_9H_{16}O_3$: C 62.77; H 9.36 %. Found: C 62.59; H 9.43 %. MS (+ESI): *m*/*z*: 196 [M^+ + Na].

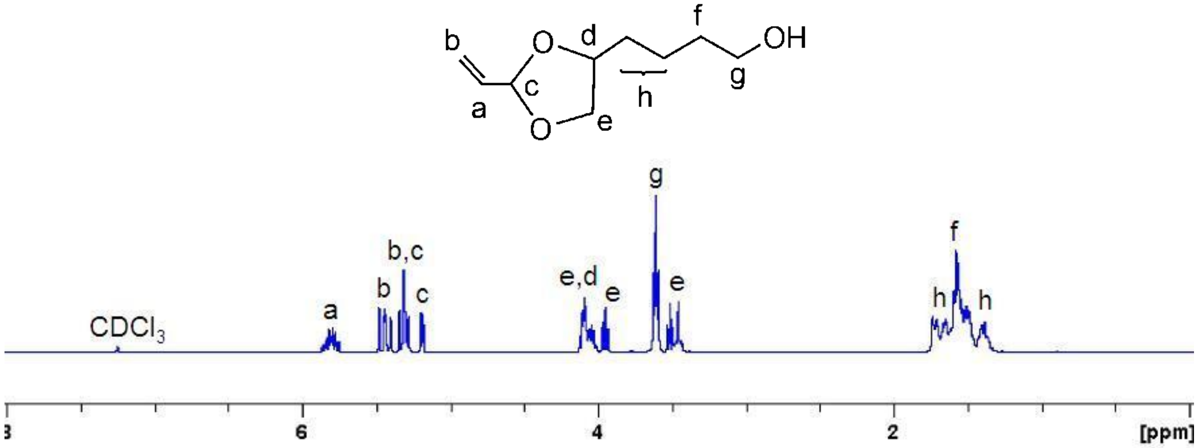

Figure S8. ^1H NMR spectrum of bifunctional monomer **10** in $CDCl_3$ (400 MHz, 298 K).

f. <u>Step-growth polymerizations of bifunctional monomers 7 - 10</u>

The polymerizations of all bifunctional monomers (**7** - **10**) were carried out similarly to those of the difunctional monomers.

A typical polymerization of the bifunctional monomer **10** is given. In the glovebox, **10** (70 mg, 0.407 mmol) was dissolved in 1,4-dioxane (0.50 mL). Catalyst **3** (2 mg, 0.002 mmol) was loaded onto the tip of a pipette and was then directly washed down a Schlenk tube equipped with a Rotaflo tap with the aforementioned monomer solution. The reaction was then sealed, brought out of the glovebox and heated at 85 °C. When the desired reaction time had been reached, the polymerization was quenched by immersing into an ice bath. The resulting polymers derived from all bifunctional monomers were then precipitated into cold methanol or hexane. The recovered polymers were then dried under vacuum prior to analysis.

(i) Characterization of ***P7*****:**

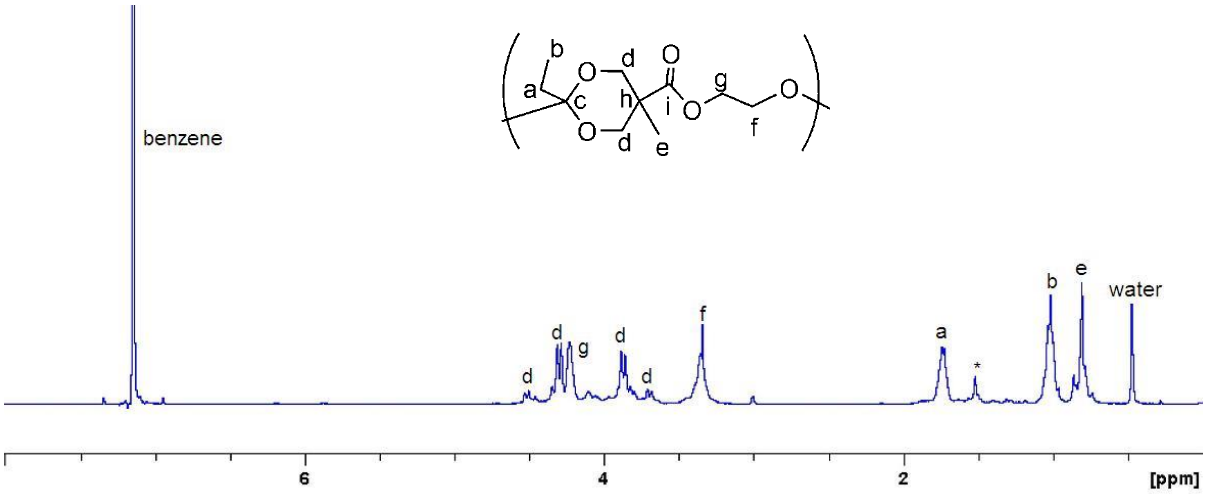

Figure S9. ^{1}H NMR spectrum of **P7** in C_6D_6 (400 MHz, 298 K).

Yield 60-73 %. ^{1}H NMR (400 MHz, C_6D_6, 298 K): δ = 4.51-3.66 (m, 4H, H_d), 4.22 (br m, 2H, H_g), 3.34 (br m, 2H, H_f), 1.73 (br m, 2H, H_a), 1.01 (br m, 3H, H_b), 1.50, 0.80 (s, 3H, H_e). ^{13}C{^{1}H} (100 MHz, C_6D_6, 298 K): δ = 174.0 (C_i), 112.7 (C_c), 65.7 (C_d), 64.1 (C_g), 60.9 (C_f), 41.8 (C_h), 28.8 (C_a), 18.3 (C_e), 7.5 (C_b). GPC ($CHCl_3$, PS standards) M_w = 8.5 kDa, $Đ_M$ = 1.53. DSC: T_g = 18.9 °C.

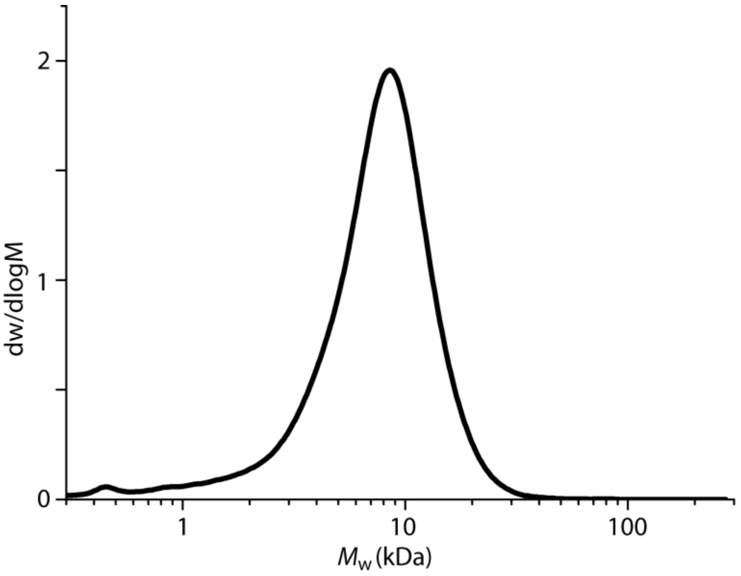

Figure S10. SEC chromatogram of **P7** ($CHCl_3$ eluent, PS standards)

(ii) Characterization of ***P8*****:**

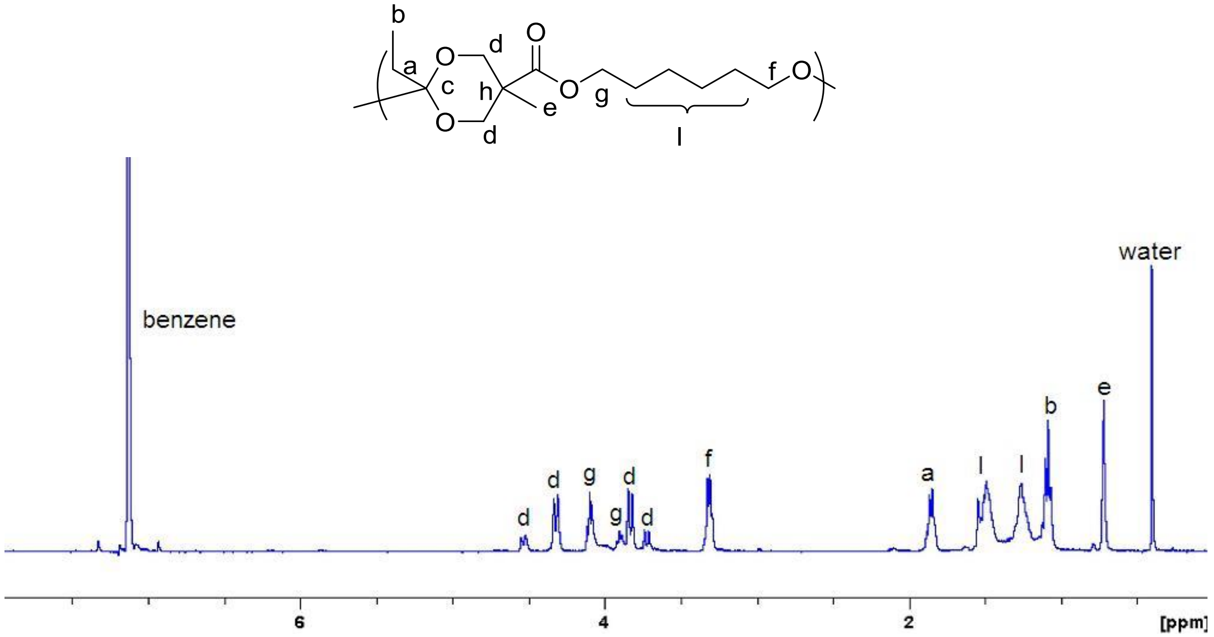

Figure S11. ^{1}H NMR spectrum of **P8** in C_6D_6 (400 MHz, 298 K).

Yield 65-70 %. ^{1}H NMR (400 MHz, C_6D_6, 298 K): δ = 4.53-3.73 (m, 4H, H_d), 4.17; 3.91 (br t, $^{3}J_{H,H}$ = 6.4, 2H, H_g), 3.32 (br m, 2H, H_f), 1.86 (br q, $^{3}J_{H,H}$ = 9.7 Hz, 2H, H_a), 1.59-1.20 (br m, 8H, H_l), 1.09 (br t, $^{3}J_{H,H}$ = 6.9 Hz, 3H, H_b), 0.72 (s, 3H, H_e). $^{13}C\{^{1}H\}$ (100 MHz, C_6D_6, 298 K): δ = 174.2 (C_i), 112.5 (C_c), 65.3 (C_d), 64.4 (C_g), 61.8 (C_f), 41.0 (C_h), 28.4 (C_a), 29.9-25.8 (C_l), 17.9 (C_e), 7.3 (C_b). GPC ($CHCl_3$, PS standards) M_w = 10.6 kDa, $Đ_M$ = 1.47. DSC: T_g = -23 °C.

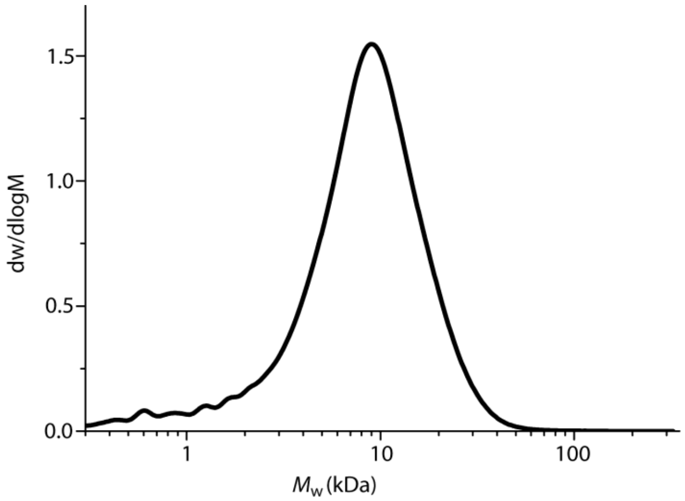

Figure S12. SEC chromatogram of **P8** ($CHCl_3$ eluent, PS standards)

(iii) Characterization of ***P9*****:**

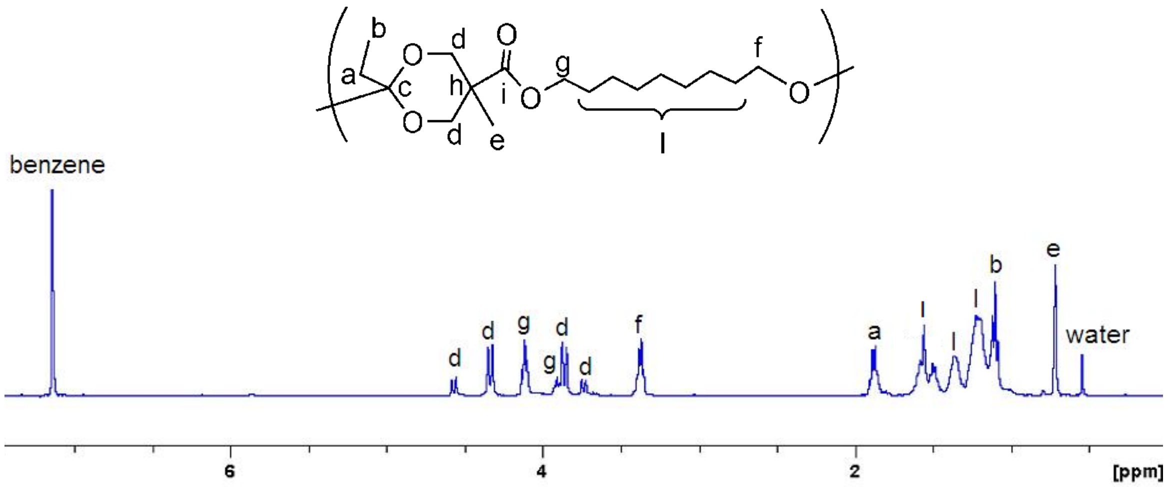

Figure S13. ^{1}H NMR spectrum of **P9** in C_6D_6 (400 MHz, 298 K).

Yield 68-75 %. ^{1}H NMR (400 MHz, C_6D_6, 298 K): δ = 4.59-3.73 (m, 4H, H_d), 4.12, 3.92 (br t, $^3J_{H,H}$ = 6.6, 2H, H_g), 3.37 (br m, 2H, H_f), 1.88 (br q, $^3J_{H,H}$ = 8.1 Hz, 2H, H_a), 1.66-1.16 (br m, 14H, H_l), 1.10 (br t, $^3J_{H,H}$ = 8.1 Hz, 3H, H_b), 0.72 (s, 3H, H_e). $^{13}C\{^1H\}$ (100 MHz, C_6D_6, 298 K): δ = 174.5 (C_i), 112.7 (C_c), 65.8-65.3 (C_d), 65.0 (C_g), 62.3 (C_f), 41.6 (C_h), 29.1 (C_a), 30.4-26.2

(C_l), 18.2 (C_e), 7.8 (C_b). SEC ($CHCl_3$, PS standards) M_w = 11.0 kDa, $Đ_M$ = 1.62. DSC: T_g = -39 °C.

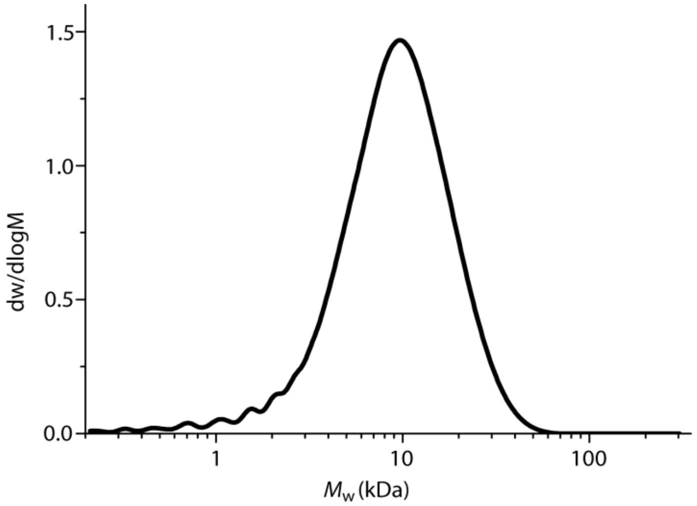

Figure S14. SEC chromatogram of **P9** ($CHCl_3$ eluent, PS standards)

*(iv) Characterization of **P10***

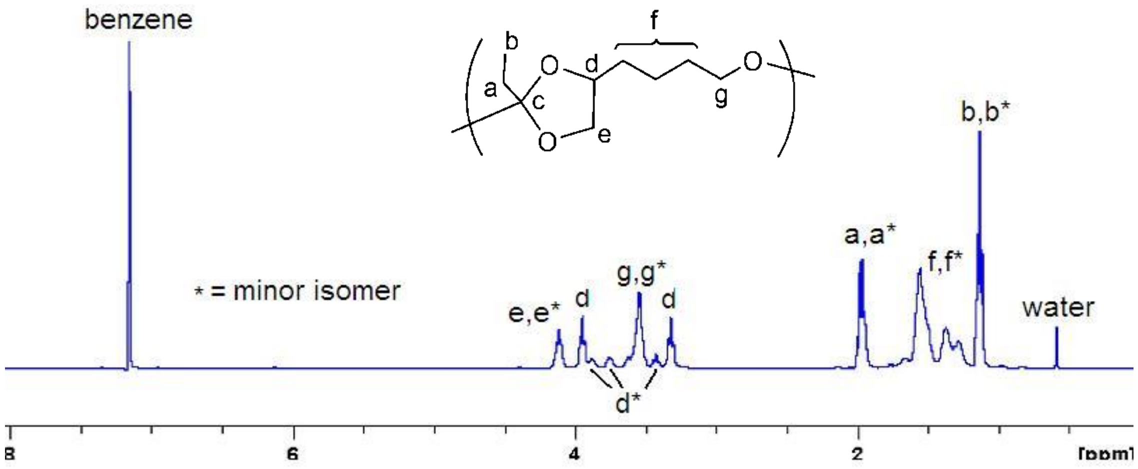

Figure S15. ^1H NMR spectrum of **P10** in C_6D_6 (400 MHz, 298 K).

Yield 75-80 %. ^1H NMR (400 MHz, C_6D_6, 298 K): δ= 4.12 (m, 1H, H_e), 3.95 (br m, 1H, H_d), 3.88 (br m , H_d, minor isomer), 3.76 (br m , H_d, minor isomer), 3.61 (br m , H_g, minor isomer), 3.54 (br m, 2H, H_g), 3.43 (br m , H_d, minor isomer), 3.32 (br m, 2H, H_d), 1.97 (br q, $^3J_{H,H}$ = 7.1 Hz, 2H, H_a), 1.80-1.20 (br m, 6H, H_f), 1.13 (br t, $^3J_{H,H}$ = 7.1 Hz, 3H, H_b). $^{13}C\{^1H\}$

(100 MHz, C_6D_6, 298 K): δ = 124.0 (C_c, minor isomer), 123.7 (C_c), 77.3 (C_e, minor isomer), 77.3 (C_e), 76.9 (C_e), 70.6 (C_d), 61.5 (C_g), 60.1 (C_g, minor isomer), 34.1, 33.5, 23.2, 22.9, 20.9, 20.6 (C_f), 30.7 (C_a, minor isomer), 30.2 (C_a), 14.3 (C_b, minor isomer), 8.8 (C_b). SEC ($CHCl_3$, PS standards) M_w = 21.2 kDa, $Đ_M$ = 1.75. DSC: T_g = -32 °C.

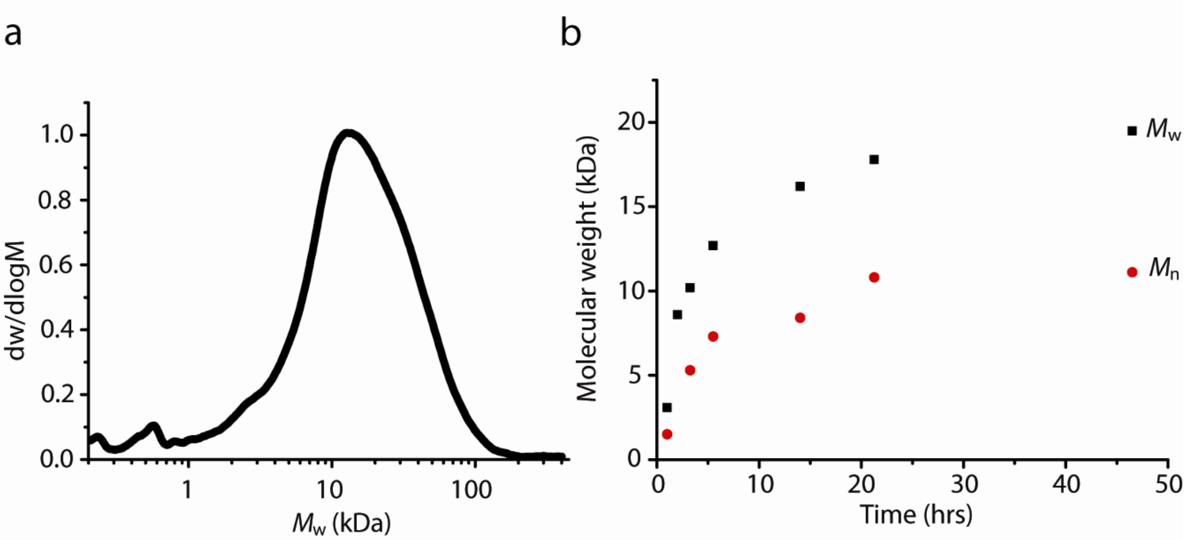

Figure S16. (a) SEC chromatogram of **P10** ($CHCl_3$ eluent, PS standards), (b) molecular weight *versus* time plot of the step-growth polymerization of **10**.

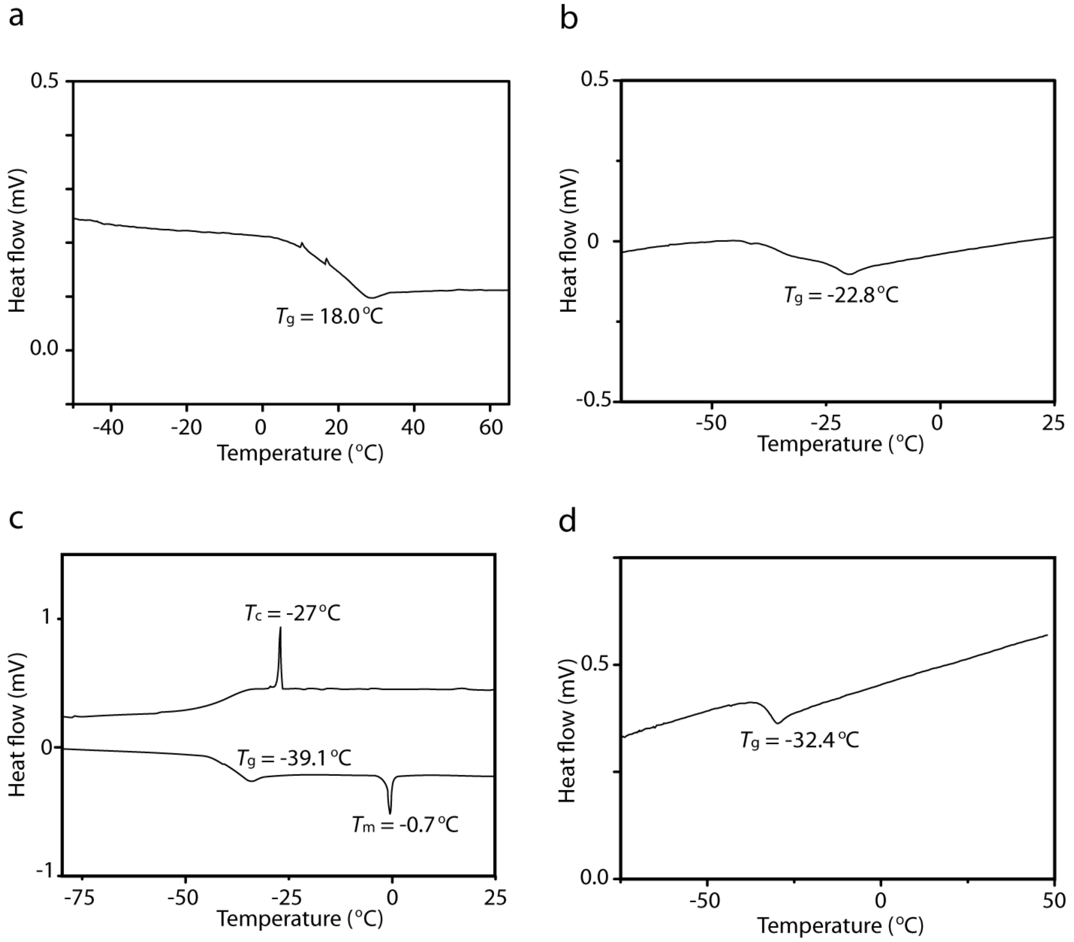

Figure S17. DSC traces of (a) **P7**, (b) **P8**, (c) **P9** and (d) **P10** obtained at a scan rate of 10 °C/min.

g. Preparation of bifunctional monomers 11-14:

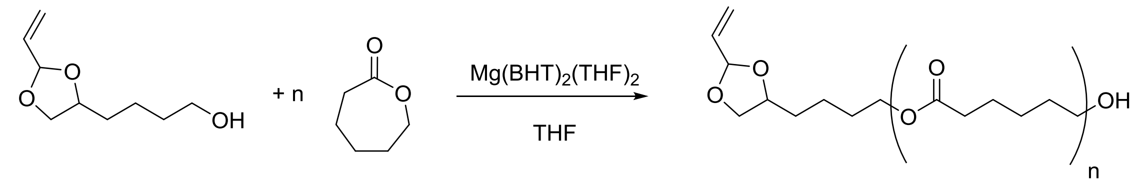

10 **11-14** (n=2, 5, 10, 20)

(i) General procedure:

In a Schlenk flask inside the glove box, $Mg(BHT)_2(THF)_2$ (1g, 1.65 mmol) is dissolved in THF (15 mL). Then, a solution of **10** (0.283 g, 1.65 mmol) in THF (3 mL) is added the Mg solution and stirred. After 20 min., the desired amount of ε-caprolactone (for **11**: 0.376 g in 2 mL THF; for **12**: 0.937g in 2 mL THF; for **13**: 1.881g in 5 mL THF; for **14**: 3.75g in 5 mL

THF) is added and the obtained solution is stirred for the desired time (30 min for **11** and **12**; 1h for **13** and **14**). The reaction was quenched with water (0.1 mL) and stirred for 20 min. Then the solvent was evaporated to dryness and the product was purified by flash column chromatography over silica gel. The impure product should be added on the column as a silica plug, otherwise the Mg salts residue blocked the column. Once added, the column is eluted with dichloromethane to remove the BHT residue and other impurities and then the product is recovered pure with CH_2Cl_2/EtOAc 1/2 as eluent (Rf (CH_2Cl_2/EtOAc 1/1) ~ 0.4). The monomers were then dissolved in toluene and dried over CaH_2 overnight. The toluene solution was filtered, and the solvent evaporated to give the pure dried bifunctional monomers **11**-**14**. The monomers were stored in the glovebox.

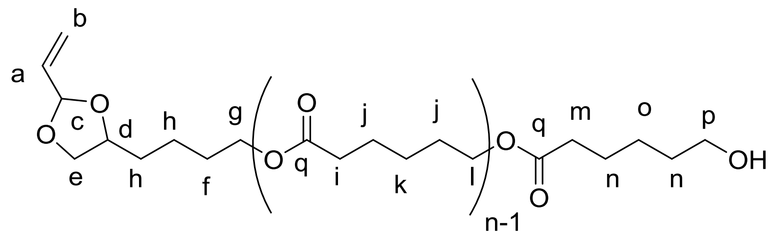

11-14 (n=2, 5, 10, 20)

*(ii) Characterization of **11**:*

11 is obtained as a colourless oil in 60 % yield:

^{1}H NMR ($CDCl_3$, 400 MHz, 298 K): δ = 5.87-5.74 (m, 1H, H_a), 5.50-5.18 (m, 3H, H_b, H_c), 4.12-3.92 (m, 4H, H_e, H_d, H_g), 4.05 (m, 3H, H_l), 3.62 (m, 2H, H_p), 3.53-3.43 (m, 1H, H_e), 2.27 (m, 5H, H_i, H_m), 1.70-1.45 (br m, 16H, H_h, H_j, H_n, H_f), 1.35 (m, 6H, H_k, H_o, H_h). $^{13}C\{^1H\}$ (100 MHz, $CDCl_3$, 298 K): δ = 173.88, 173.64, 173.58 (C_q), 134.82, 134.78 (C_a), 120.55, 120.50, 120.45, 119.80, 119.74, 119.70 (C_b), 104.15, 104.11, 103.39, 103.35 (C_c), 77.16, 76.92, 76.18, 76.06, 76.04 (C_d), 70.4, 70.36, 69.79, 69.75 (C_e), 64.24, 64.20, 64.16 (C_l,C_g), 62.59 (C_p), 34.32 (H_m), 34.21 (C_i), 33.15, 33.04, 32.94, 32.82, 32.65, 32.42 (C_f,C_h), 28.66 (C_n), 28.43 (C_j), 25.65, 25.62, 25.40 (C_k,C_o), 24.78 (C_n), 24.67 (C_j), 22.44, 22.39, 22.17, 22.14 (C_h).

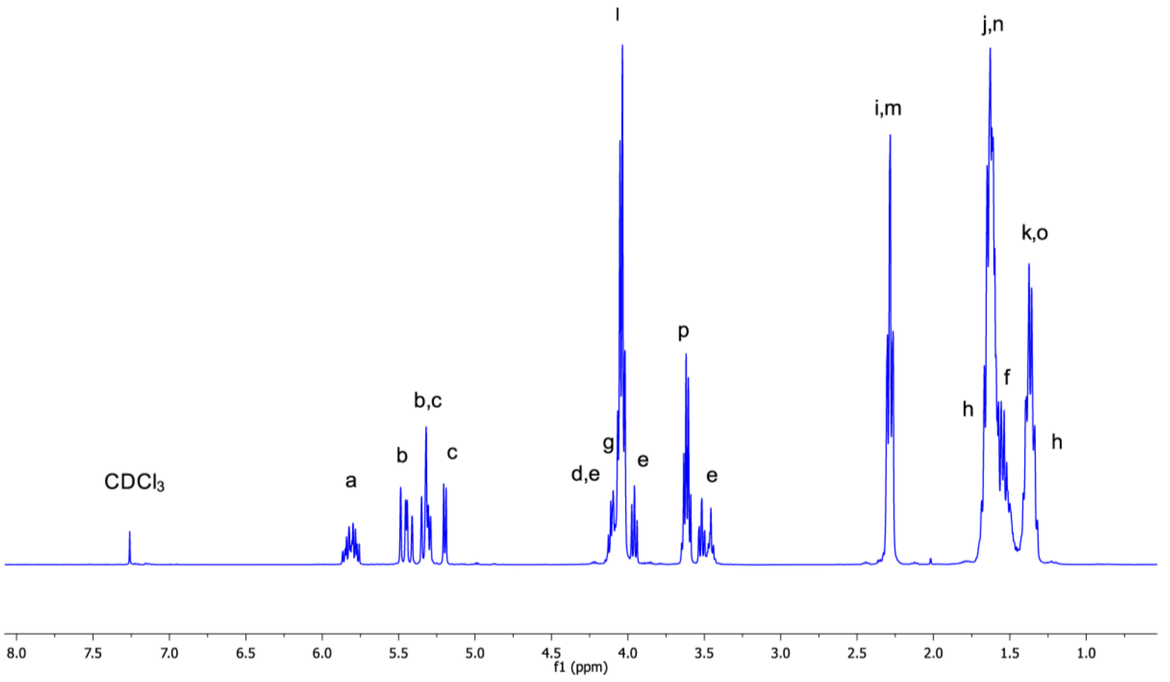

Figure S18. ^{1}H NMR spectrum of **11** in $CDCl_3$ (400 MHz, 298 K).

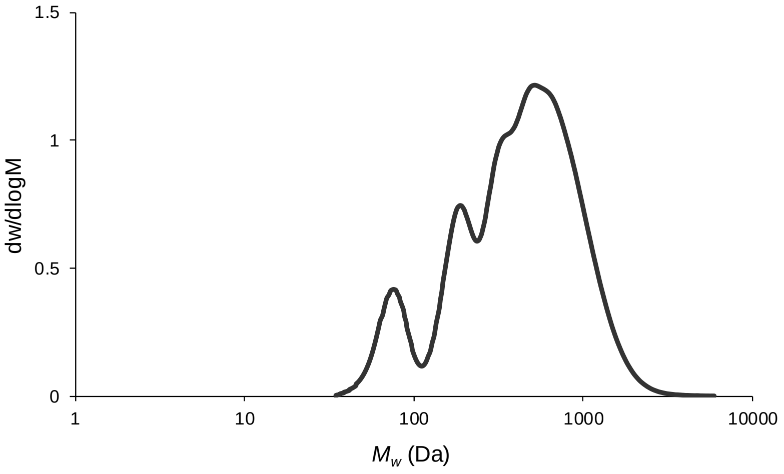

Figure S19. SEC chromatogram of **11** (THF eluent, PS standards).

*(iii) Characterization of **12**:*

12 is obtained as a white sticky solid in 58 % yield:

^{1}H NMR ($CDCl_3$, 400 MHz, 298 K): δ = 5.87-5.74 (m, 1H, H_a), 5.50-5.18 (m, 3H, H_b, H_c), 4.12-3.92 (m, 4H, H_e, H_d,H_g), 4.05 (t, J = 6.6 Hz, 10H, H_l), 3.62 (m, 2H, H_p), 3.53-3.43 (m, 1H, H_e),

2.27 (t, J = 7.4 Hz, 12H, $H_{i,}H_m$), 1.70-1.45 (br m, 32H, H_h, H_j, H_n, H_f), 1.35 (m, 14H, H_k, H_o, H_h). $^{13}C\{^1H\}$ (100 MHz, $CDCl_3$, 298 K): δ = 173.82, 173.68, 173.63 (C_q), 134.85 (C_a), 120.47, 119.72 (C_b), 104.14, 103.38 (C_c), 76.92, 76.04 (C_d), 70.35, 69.75 (C_e), 64.22, 64.18 (C_l,C_g), 62.66 (C_p), 34.32 (H_m), 34.20 (C_i), 33.04, 32.82, 32.42 (C_f,C_h), 28.66 (C_n), 28.43 (C_j), 25.65, 25.40 (C_k,C_o), 24.78 (C_n), 24.67 (C_j), 22.44, 22.38 (C_h).

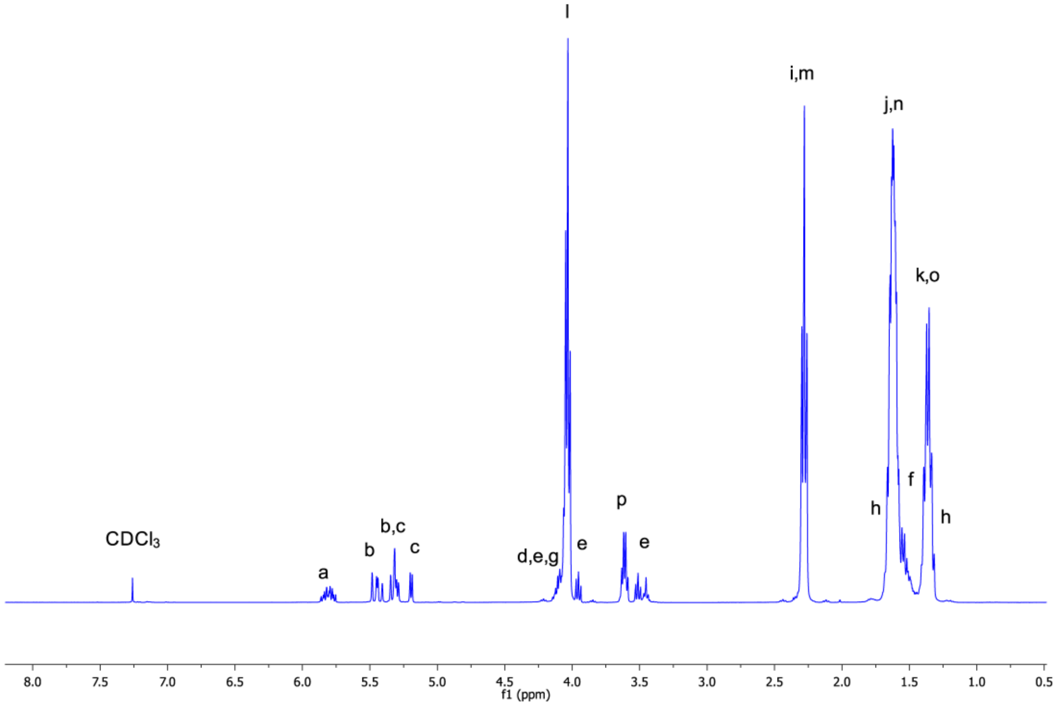

Figure S20. ^1H NMR spectrum of **12** in $CDCl_3$ (400 MHz, 298 K).

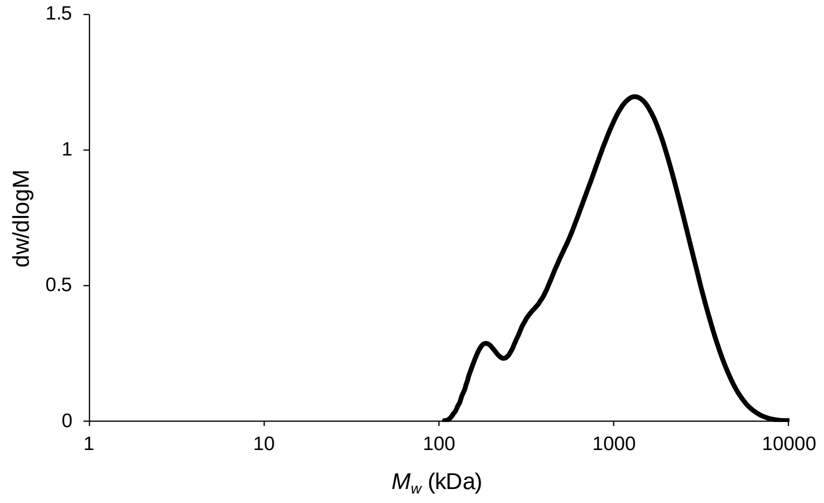

Figure S21. SEC chromatogram of **12** (THF eluent, PS standards).

*(iv) Characterization of **13**:*

13 is obtained as a white solid in 63% yield:

^{1}H NMR ($CDCl_3$, 400 MHz, 298 K): δ = 5.87-5.74 (m, 1H, H_a), 5.50-5.18 (m, 3H, H_b, H_c), 4.12-3.92 (m, 4H, H_e, H_d,H_g), 4.05 (t, *J* = 6.6 Hz, m, 22H, H_l), 3.62 (m, 2H, H_p), 3.53-3.43 (m, 1H, H_e), 2.27 (t, *J* = 7.4 Hz, 24H, H_i,H_m), 1.70-1.45 (br m, 56H, H_h, H_j, H_n, H_f), 1.35 (m, 26H, H_k, H_o, H_h). ^{13}C{^{1}H} (100 MHz, $CDCl_3$, 298 K): δ = 173.82, 173.68, 173.62 (C_q), 134.86 (C_a), 120.47, 119.72 (C_b), 104.15, 103.39 (C_c), 76.92, 76.04 (C_d), 70.35, 69.75 (C_e), 64.22, 64.18 (C_l,C_g), 62.66 (C_p), 34.32 (H_m), 34.21 (C_i), 33.05, 32.83, 32.43 (C_f,C_h), 28.67 (C_n), 28.44 (C_j), 25.62, 25.41 (C_k,C_o), 24.78 (C_n), 24.67 (C_j), 22.45, 22.39 (C_h).

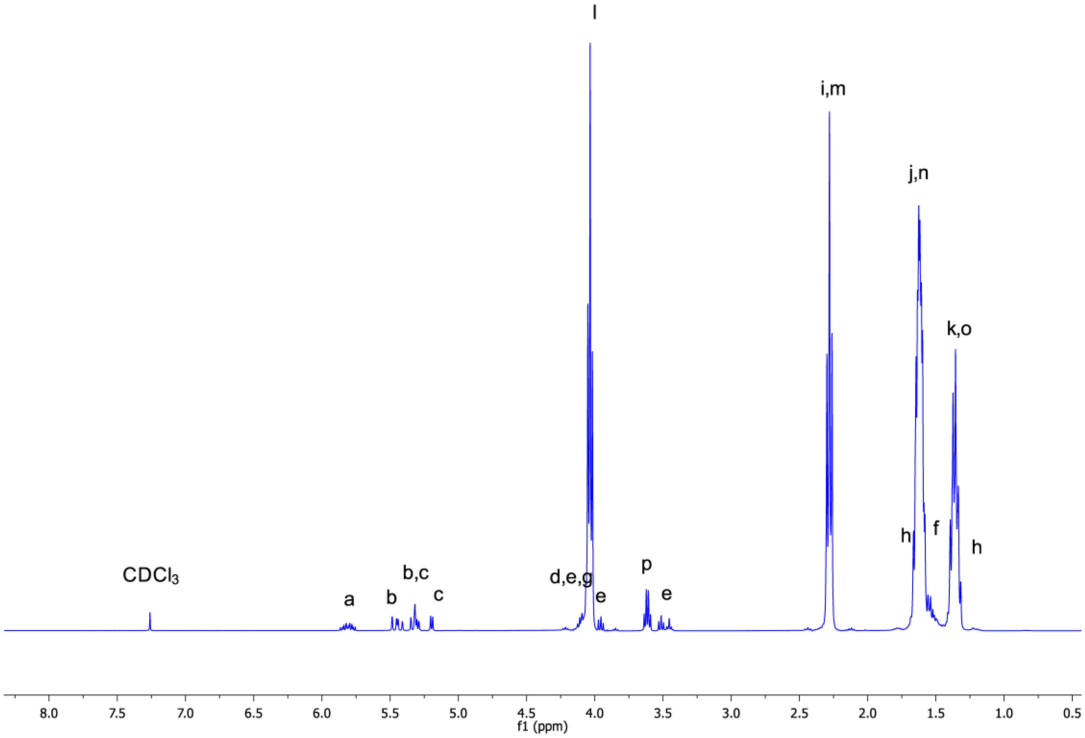

Figure S22. ^{1}H NMR spectrum of **13** in $CDCl_3$ (400 MHz, 298 K).

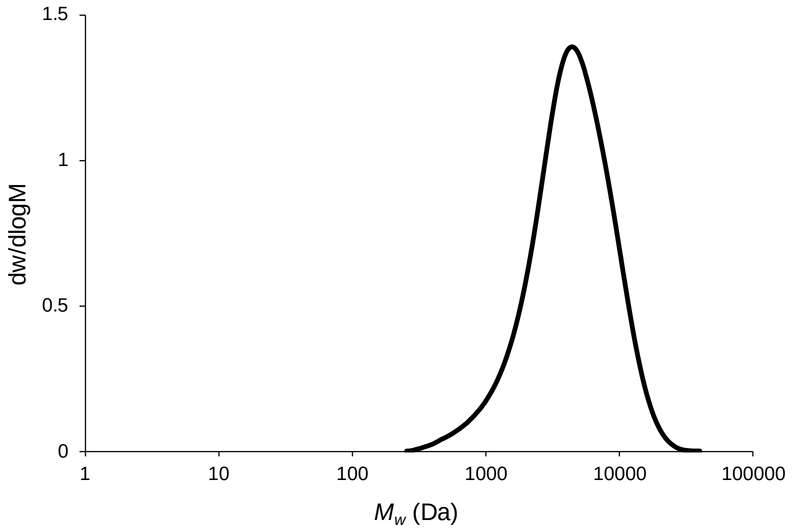

Figure S23. SEC chromatogram of **13** (THF eluent, PS standards).

*(v) Characterization of **14**:*

14 is obtained as a white powder in 67 % yield:

^{1}H NMR ($CDCl_3$, 400 MHz, 298 K): δ = 5.87-5.74 (m, 1H, H_a), 5.50-5.18 (m, 3H, $H_{b,}$ H_c), 4.12-3.92 (m, 4H, $H_{e,}$ $H_{d,}H_g$), 4.05 (t, J = 6.6 Hz,, 42H, H_l), 3.62 (m, 2H, H_p), 3.53-3.43 (m, 1H, H_e), 2.27 (t, J = 7.4 Hz, 44H, $H_{i,}H_m$), 1.70-1.45 (br m, 96H, H_h, H_j, H_n, H_f), 1.35 (m, 46H, H_k, H_o, H_h). ^{13}C{^{1}H} (100 MHz, $CDCl_3$, 298 K): δ = 173.57 (C_q), 134.88 (C_a), 120.49, 119.74 (C_b), 104.17, 103.41 (C_c), 76.94, 76.06 (C_d), 70.38, 69.78 (C_e), 64.24, 64.20 (C_l,C_g), 62.71 (C_p), 34.34 (H_m), 34.22 (C_i), 33.07, 32.84, 32.45 (C_f,C_h), 28.68 (C_n), 28.46 (C_j), 25.64, 25.42 (C_k,C_o), 24.80 (C_n), 24.68 (C_j), 22.46, 22.41 (C_h).

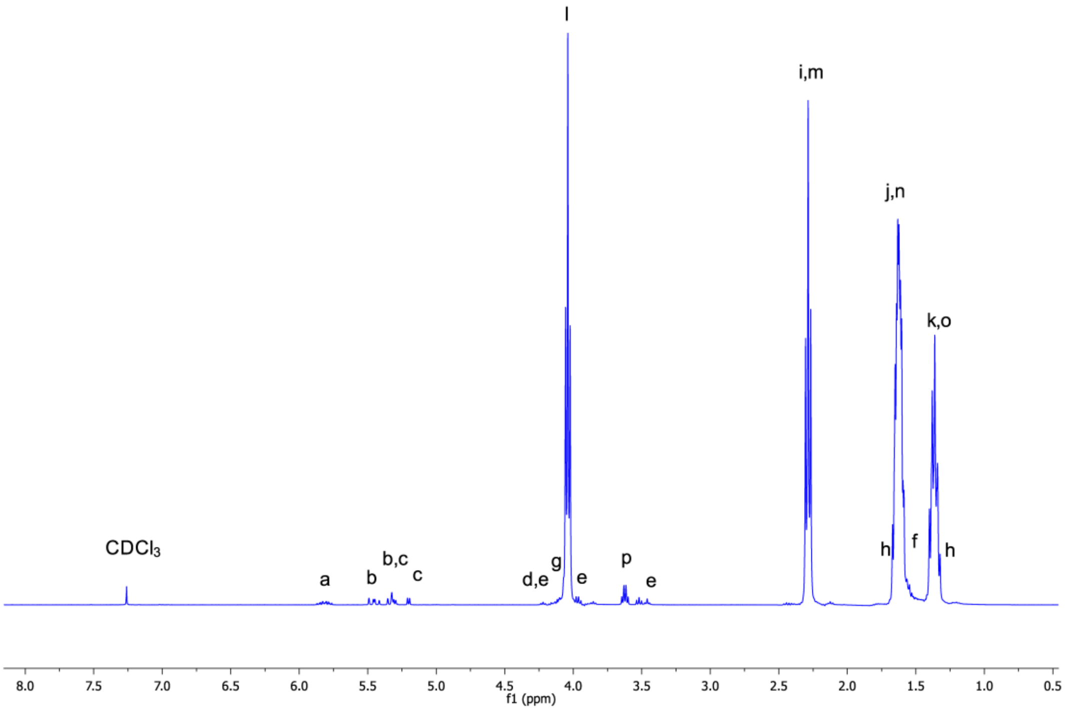

Figure S24. ^{1}H NMR spectrum of **14** in $CDCl_3$ (400 MHz, 298 K).

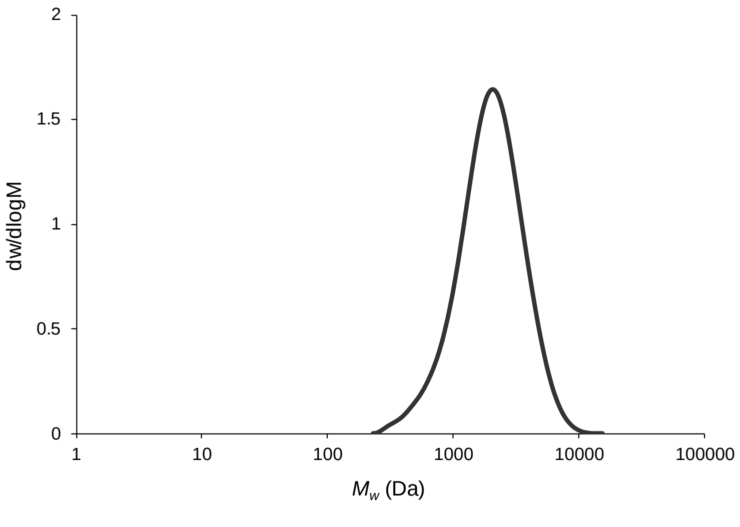

Figure S25. SEC chromatogram of **14** (THF eluent, PS standards).

Table S1. Data analysis for **11-14**.

	M_{calc} (kDa)	M_{NMR} (kg.mol^{-1})^{a}	M_w (kDa)^{b}	M_n (kDa)^{b}	$Đ_M$^{b}
**11**	0.40	0.45 (DP 2.5)	0.53	0.27	1.95
**12**	0.74	0.90 (DP 6.5)	1.38	0.73	1.89
**13**	1.31	1.60 (DP 12.5)	2.31	1.63	1.42
**14**	2.45	2.73 (DP 22.5)	5.33	3.21	1.65

a) determined by ^{1}H NMR in $CDCl_3$ (comparing chain-end CH_2 (p) and CH_2 (m+i)); b) determined by SEC analysis in THF (vs PS standards).

h. Step-growth polymerisations of monomers 11-14.

(i) General procedure

In the glovebox, the monomer (**11**: 120 mg; **12**: 200mg; **13**: 262mg; **14**: 490 mg) was dissolved in the desired solvent 1,4-dioxane (**11** and **12** in 0.50 mL) or toluene (**13** in 0.8 mL; **14** in 1.5 mL). [RuHCl(PPh_3)$_3$].toluene catalyst (2 mg, 0.002 mmol) was weighted in a vial and the vial was "washed" down with the aforementioned solution and introduced in Schlenk ampoule equipped with a Rotaflo tap. The reaction was then sealed, brought out of the glovebox and heated at 85 °C. When the desired reaction time was reached, the polymers were directly precipitated from hexane (**P11** and **P12**) or MeOH (**P13** and **P14**). The recovered polymers were then dried under vacuum prior to analysis (yield 60-70%).

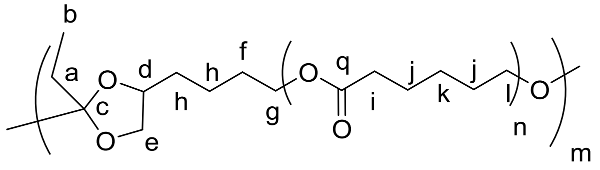

P11-P14 (**P11**: n=2; **P12**: n=5; **P13**: n=10; **P14**: n=20)

*(ii) Characterization of **P11**:*

^{1}H NMR (400 MHz, C_6D_6, 298 K): δ = 4.08 (m, 1H, H_e), 3.97 (m, 3H, $H_{l,}$), 3.96 (m , 2H, H_g), 3.95 (m, H_d), 3.75 (m, H_d), 3.52 (m , 2H H_l in α of orthoester), 3.40 (m, H_d), 3.28 (m, H_d), 2.16 (m, 2H H_i in α of orthoester), 2.10 (m, 3H, H_i), 1.94 (m, 2H, H_a), 1.63 (m, 2H H_j in α of

orthoester), 1.54 (m, 3H, H_j), 1.39 (m, 5H, H_j), 1.50-1.15 (m, 6H, H_f), 1.17 (m, 5H, H_k), 1.11 (m, 3H, H_b). $^{13}C\{^1H\}$ (100 MHz, C_6D_6, 298 K): Characteristic signals: δ = 172.98, 172.79 (C_q), 123.97, 123.66 (C_c), 8.73 (C_b). SEC (THF, PS standards) M_w = 12.2 kDa, $Đ_M$ = 1.82.

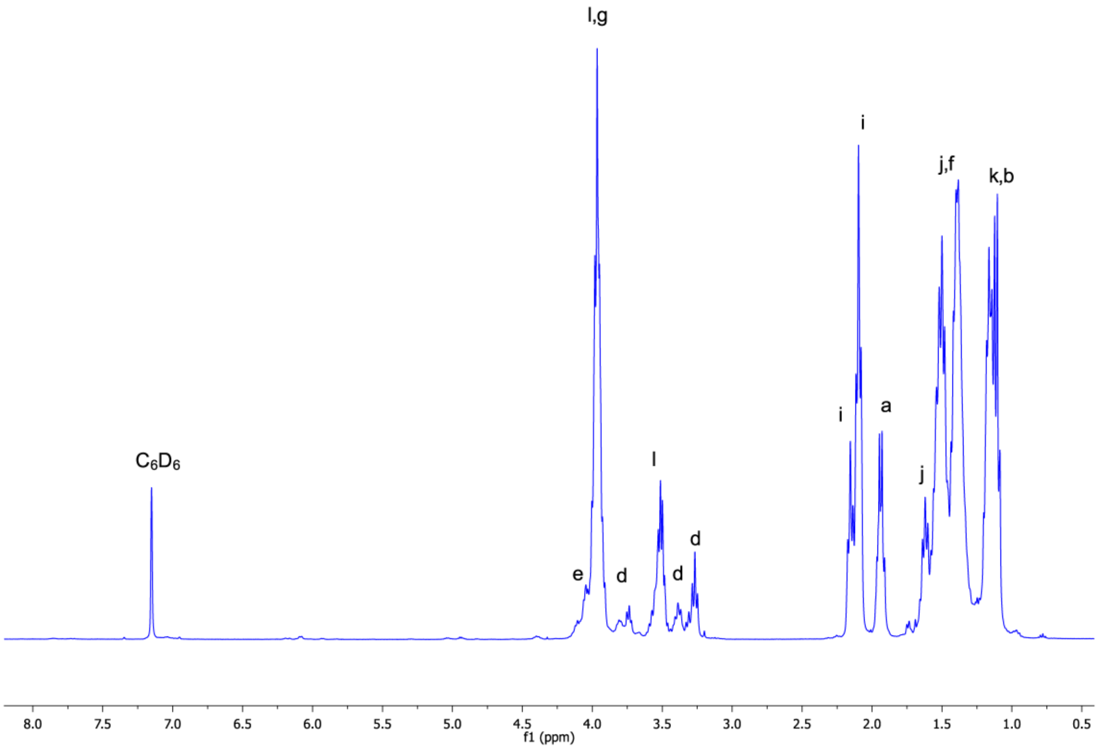

Figure S26. ^1H NMR spectrum of **P11** in C_6D_6 (400 MHz, 298 K).

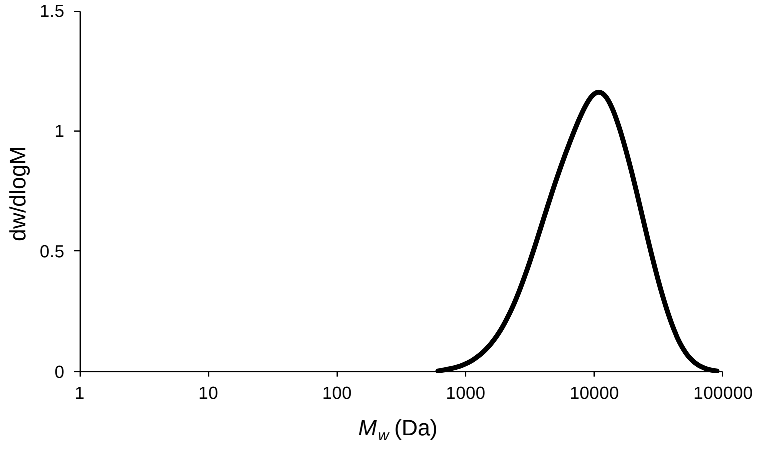

Figure S27. SEC chromatogram of **P11** (THF eluent, PS standards).

(iii) Characterization of ***P12*****:**

^{1}H NMR (400 MHz, C_6D_6, 298 K): δ = 4.07 (m, 1H, H_e), 3.97 (t, 10H, *J* = 6.6 Hz, $H_{l,}$), 3.96 (m , 2H, H_g), 3.95 (m, H_d), 3.78 (m, H_d), 3.52 (m , 2H H_l in α of orthoester), 3.40 (m, H_d), 3.28 (m, H_d), 2.14 (m, 2H H_i in α of orthoester), 2.10 (t, 10H, *J* = 7.4 Hz, H_i), 1.94 (m, 2H, H_a), 1.63 (m, 2H, H_j in α of orthoester), 1.51 (m, 10H, H_j), 1.41 (m, 12H, $H_{j'}$), 1.5-1.1 (m, 6H, H_f), 1.17 (m, 12H, H_k), 1.08 (m, 3H, H_b). Characteristic signals: δ = 172.98, 172.78 (C_q), 123.65 (C_c), 8.73, 8.69 (C_b). SEC (THF, PS standards) M_w = 25.2 kDa, $Đ_M$ = 1.88. DSC: T_g = -57.3 °C, T_m = 41.3°C, T_c = 0.5°C.

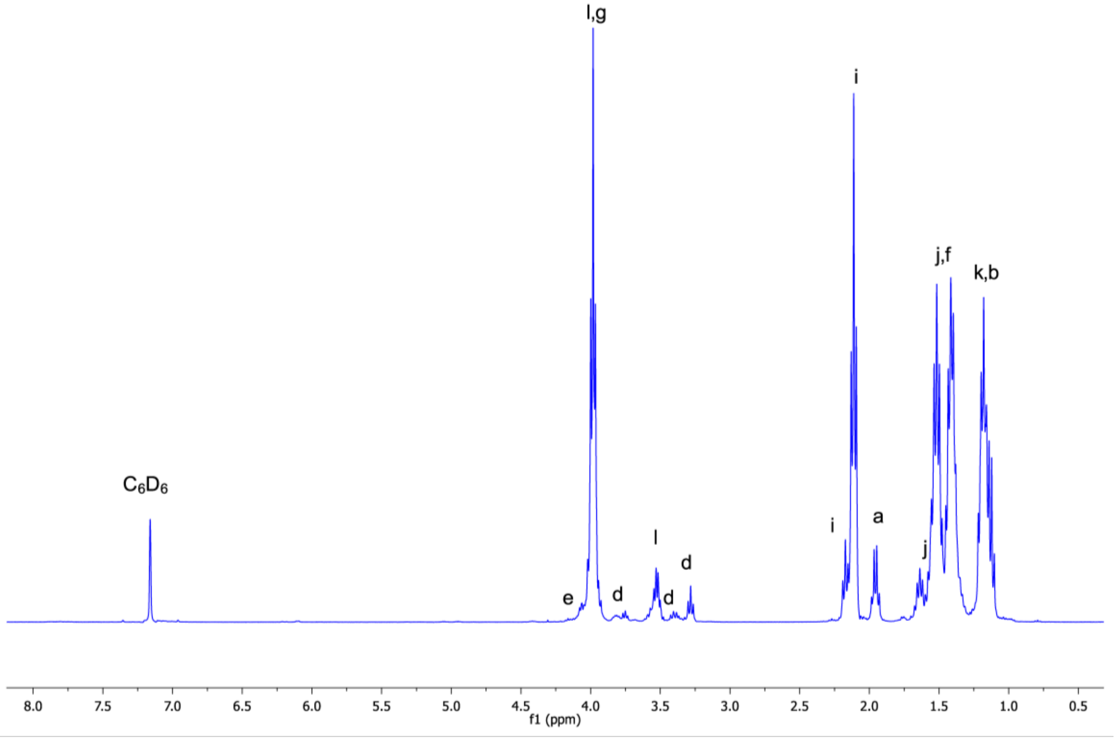

Figure S28. ^{1}H NMR spectrum of **P12** in C_6D_6 (400 MHz, 298 K).

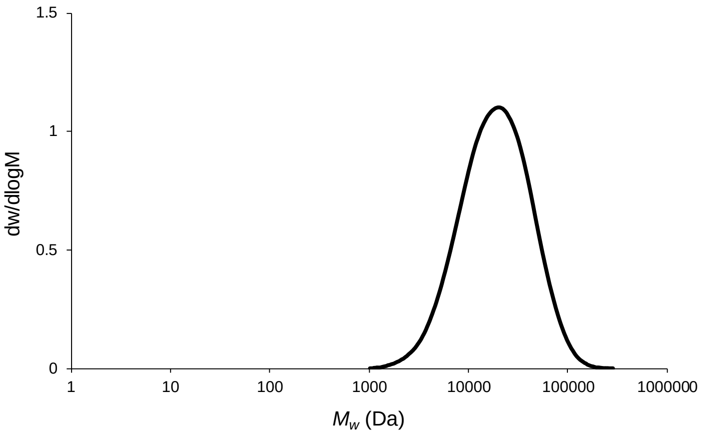

Figure S29. SEC chromatogram of **P12** (THF eluent, PS standards).

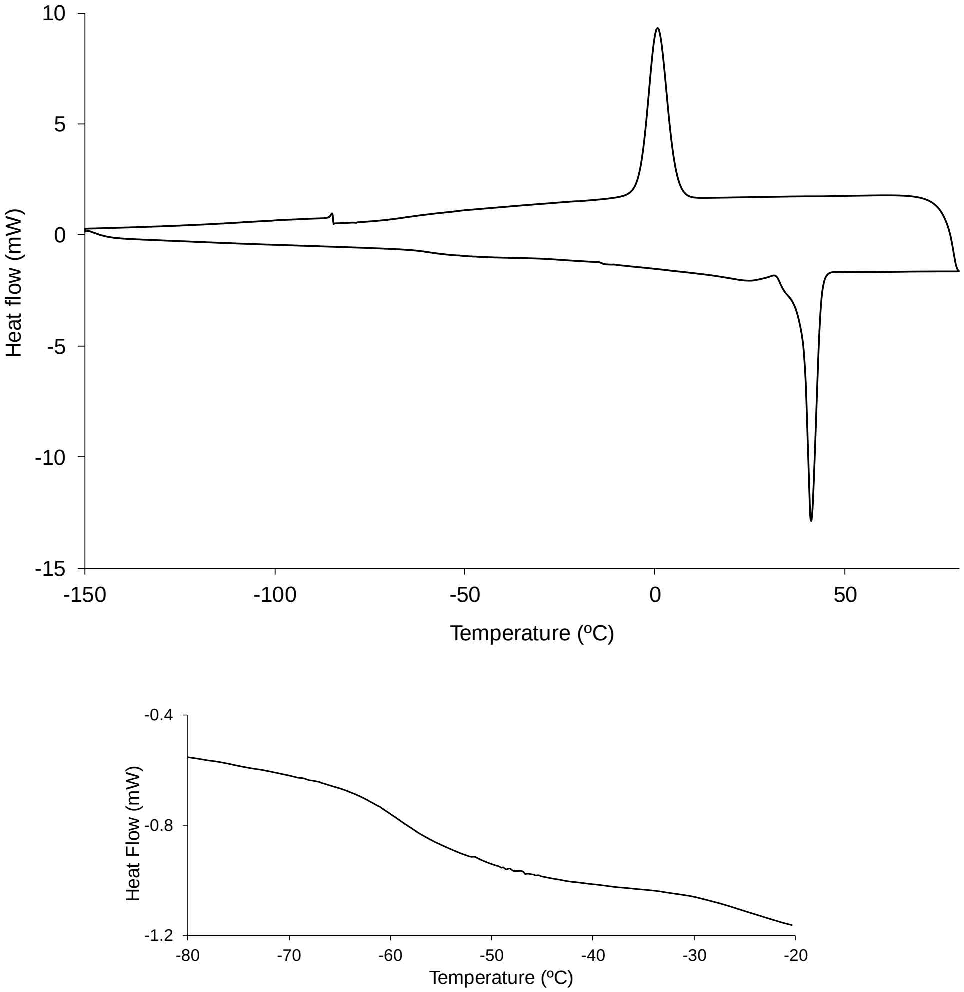

Figure S30. DSC trace of **P12** (**up:** full trace, **down:** zoom from -80 to -20°C) obtained at a scan rate of 10 °C/min.

*(iv) Characterization of **P13***:

^{1}H NMR (400 MHz, C_6D_6, 298 K): δ = 4.07 (m, 1H, H_e), 3.97 (t, 22H, J = 6.6 Hz, $H_{l,}$), 3.96 (m , 2H, H_g), 3.95 (m, H_d), 3.78 (m, H_d), 3.52 (m , 2H H_l in α of orthoester), 3.40 (m, H_d), 3.28 (m, H_d), 2.14 (m, 2H H_i in α of orthoester), 2.10 (t, 22H, J = 7.4 Hz, H_i), 1.94 (m, 2H, H_a), 1.63 (m, 2H H_j in α of orthoester), 1.51 (m, 22H, H_j), 1.41 (m, 24H, $H_{j'}$), 1.5-1.1 (m, 6H, H_f), 1.17 (m, 24H, H_k), 1.08 (m, 3H, H_b). ^{13}C{^{1}H} (100 MHz, C_6D_6, 298 K): Characteristic signals: δ = 172.98, 172.78 (C_q), 123.65 (C_c), 8.73, 8.69 (C_b). SEC (THF, PS standards) M_w = 43.2 kDa, $Đ_M$ = 1.75. DSC: T_g = -59.1 °C, T_m = 51.0°C, T_c = 9.0°C.

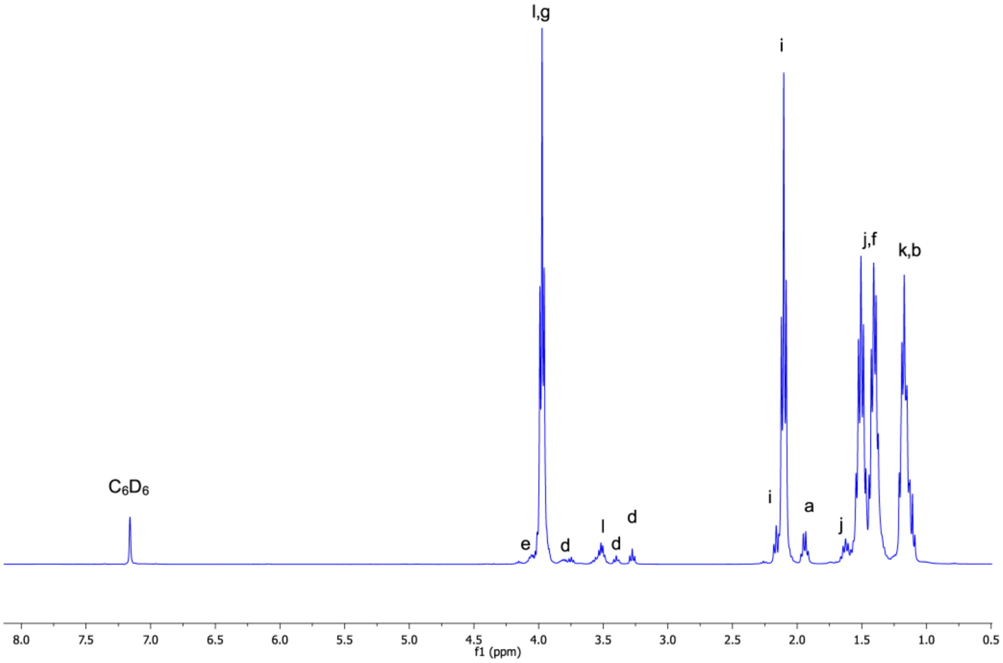

Figure S31. ^{1}H NMR spectrum of **P13** in C_6D_6 (400 MHz, 298 K).

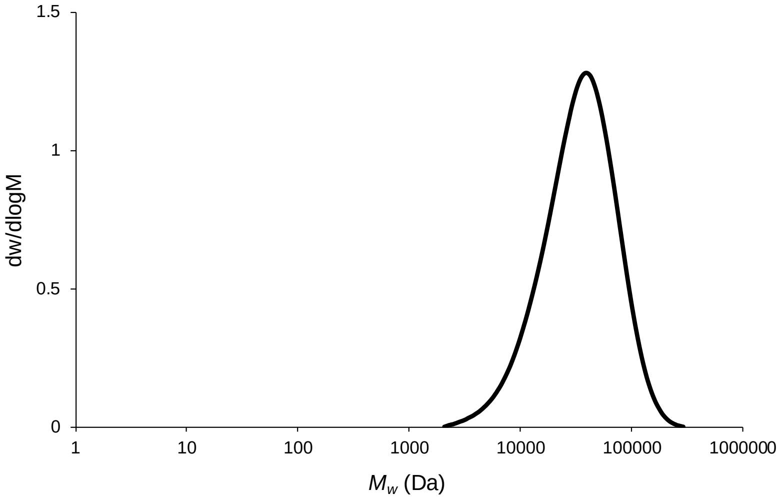

Figure S32. SEC chromatogram of **P13** (THF eluent, PS standards).

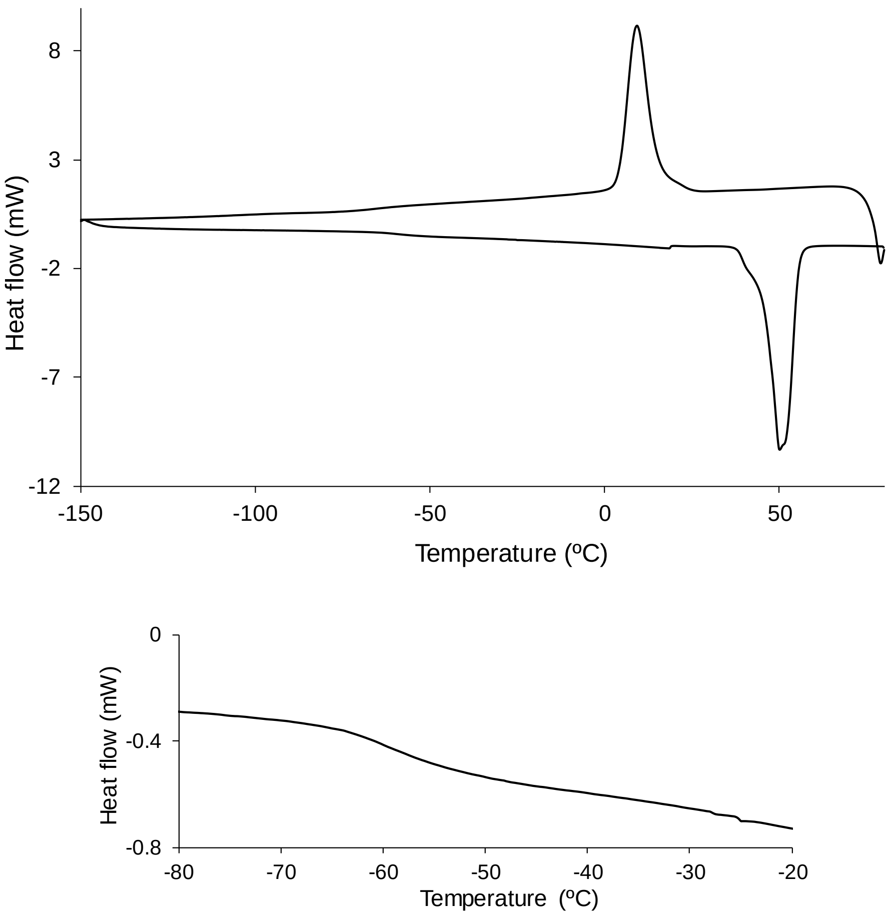

Figure S33. DSC trace of **P13** (**up:** full trace, **down:** zoom from -80 to -20°C) **P10** obtained at a scan rate of 10 °C/min.

*(v) Characterization of **P14***:

^{1}H NMR (400 MHz, C_6D_6, 298 K): δ= 4.07 (m, 1H, H_e), 3.97 (t, 42H, J = 6.6 Hz, $H_{l,}$), 3.96 (m , 2H, H_g), 3.95 (m, H_d), 3.78 (m, H_d), 3.52 (m , 2H H_l in α of orthoester), 3.40 (m, H_d), 3.28 (m, H_d), 2.14 (m, 2H H_i in α of orthoester), 2.10 (t, 42H, J = 7.4 Hz, H_i), 1.94 (m, 2H, H_a), 1.63 (m, 1.5H H_j in α of orthoester), 1.51 (m, 42H, H_j), 1.41 (m, 44H, $H_{j'}$), 1.5-1.1 (m, 6H, H_f), 1.17 (m, 44H, H_k), 1.08 (m, 3H, H_b). $^{13}C\{^1H\}$ (100 MHz, C_6D_6, 298 K): Characteristic signals: δ= 172.78 (C_q), 123.66 (C_c), 8.73 (C_b). SEC (THF, PS standards) M_w = 48.8 kDa, $Đ_M$ = 2.28. DSC: T_g = -58.9 °C, T_m = 61.1°C, T_c = 18.8°C.

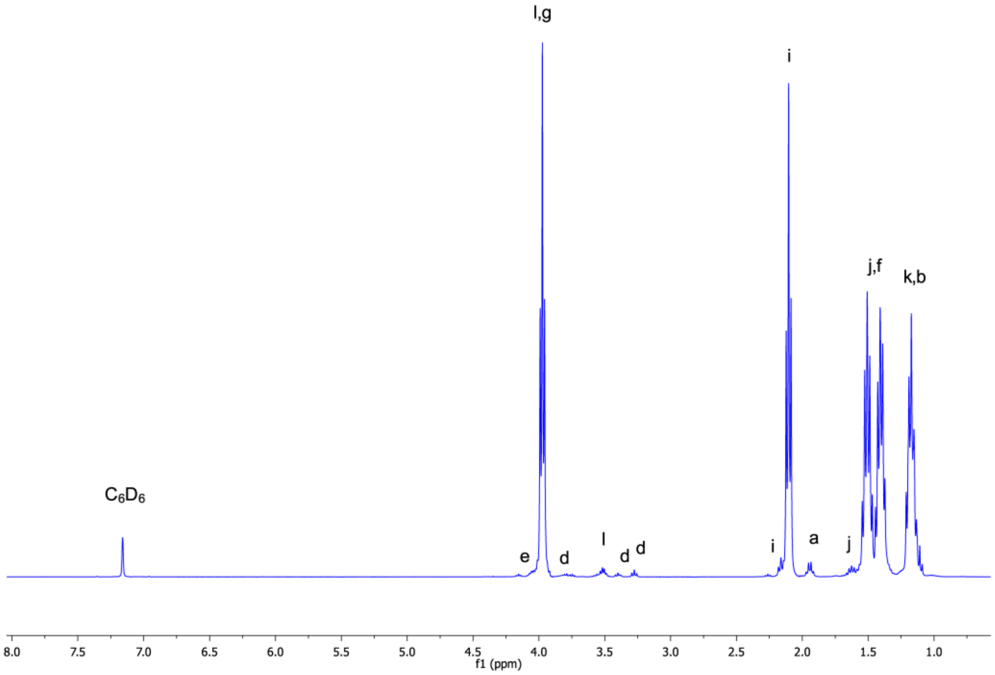

Figure S34. ^{1}H NMR spectrum of **P14** in C_6D_6 (400 MHz, 298 K).

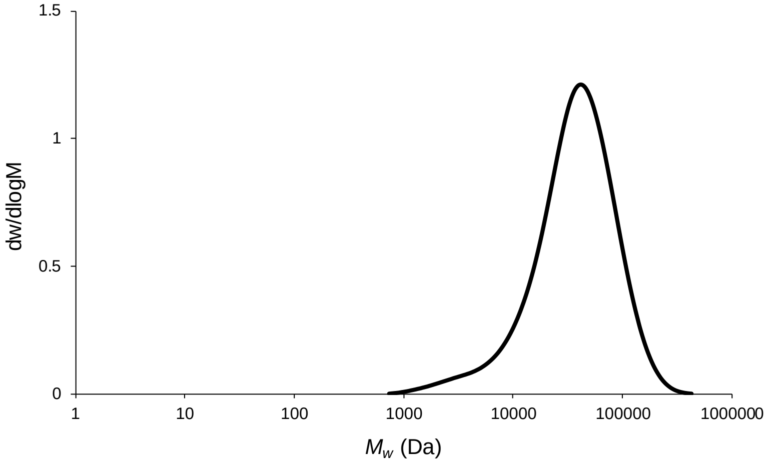

Figure S35. SEC chromatogram of **P14** (THF eluent, PS standards).

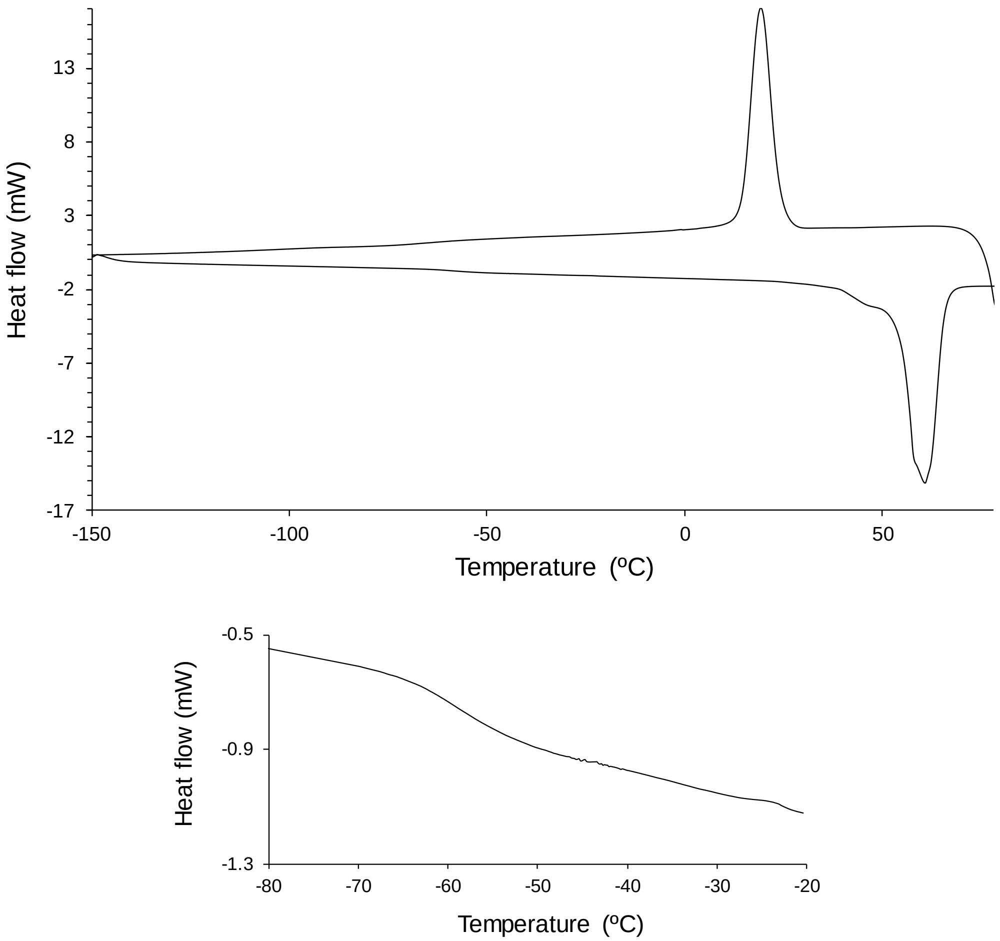

Figure S36. DSC trace of **P14** (**up:** full trace, **down:** zoom from -80 to -20°C) obtained at a scan rate of 10 °C/min.

Table S2. Synthesis of **P11**-**P14**, reproducibility tests[a)]

Entry	Polymer	Solvent	t (h)	Yield (%)	M_w (kDa)[b]	M_n (kDa)[b]	$Đ_M$[b]
1	**P11**	dioxane	48	57	9350	5600	1.79
2	**P11**	dioxane	48	62	12300	6700	1.82
3	**P12**	dioxane	48	61	27300	14000	1.94
4	**P12**	dioxane	48	63	28800	15300	1.88*
5	**P12**	dioxane	48	64	25200	13400	1.88*
6	**P13**	toluene	48	66	39200	23700	1.94
7	**P13**	toluene	48	65	37800	20900	1.80*
8	**P13**	toluene	48	67	43200	24600	1.75*
9	**P14**	toluene	48	68	44800	20000	2.23
10	**P14**	toluene	72	69	46300	20300	2.27*
11	**P14**	toluene	48	73	48800	21300	2.28*

a) Polymerization conditions: 85ºC, Ru (~1 mol%); b) SEC chromatography in THF (vs PS standard). * precipitated in MeOH.

i. Synthesis of orthoester-functionalized poly(carbonate)

(i) Synthesis of (2-vinyl-1,3-dioxane-5,5-diyl)dimethanol

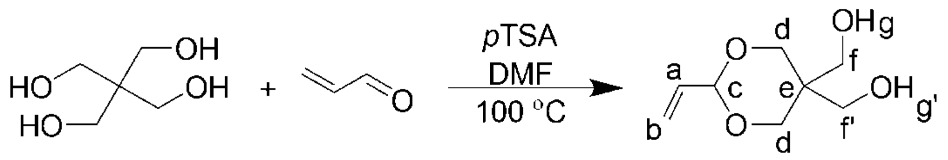

To a round bottom flask was added pentaerythritol (50 g, 0.367 mol, 1 eq) and DMF (250 mL). This was heated at 120 °C until complete dissolution, before being allowed to cool 100 °C. *p*TSA (2.5 g, 0.013 mol) was added followed by the slow addition of acrolein (25 mL, 0.367 mol, 1 eq). The resulting reaction mixture was then heated at 100 °C for 3 h after which the reaction solution was allowed to cool and volatiles removed under reduced pressure. The crude mixture was extracted with CH_2Cl_2 and the unreacted starting material removed by filtration. Evaporation of solvent gave a yellow oil, which was purified by passing through a plug of silica (CH_2Cl_2 and diethyl ether eluent respectively) to obtain the desired product. Recrystallization in toluene yielded white crystals (28.9 g, 45%).

^1H NMR (400 MHz, $(CD_3)_2SO$, 298 K): δ = 5.77 (ddd, $^3J_{H,H}$ = 17.4, 10.7, 4.5 Hz, 1H, H_a), 5.36 (d, $^3J_{H,H}$ = 17.4 Hz, 1H, H_b), 5.22 (d, $^3J_{H,H}$ = 10.7 Hz, 1H, H_b), 4.83 (d, $^3J_{H,H}$ = 4.5 Hz, 1H, H_c), 4.56 (t, $^3J_{H,H}$ = 5.3 Hz, 1H, H_g), 4.47 (t, $^3J_{H,H}$ = 5.2 HZ, 1H, $H_{g'}$), 3.79 (d, $^3J_{H,H}$ = 11.5 Hz, 2H, H_d), 3.62 (d, $^3J_{H,H}$ = 11.5 Hz, 2H, H_d), 3.56 (d, $^3J_{H,H}$ = 5.4 Hz, 2H, H_f), 3.18 (d, $^3J_{H,H}$ = 5.2 Hz, 2H, $H_{f'}$). $^{13}C\{^1H\}$ NMR (100 MHz, $(CD_3)_2SO$, 298 K): δ = 135.4 (C_a), 118.05 (C_b), 99.9 (C_c), 68.6 (C_d), 61.1 ($C_{f,f'}$), 59.47 (C_e). Anal Calcd for $C_8H_{14}O_4$: C 55.16; H 8.10 %. Found: C 55.26; H 8.10 %. HRMS Calcd for $C_8H_{14}O_4Na$: 197.0784; found: 196.0782. Mp: 78-79 °C.

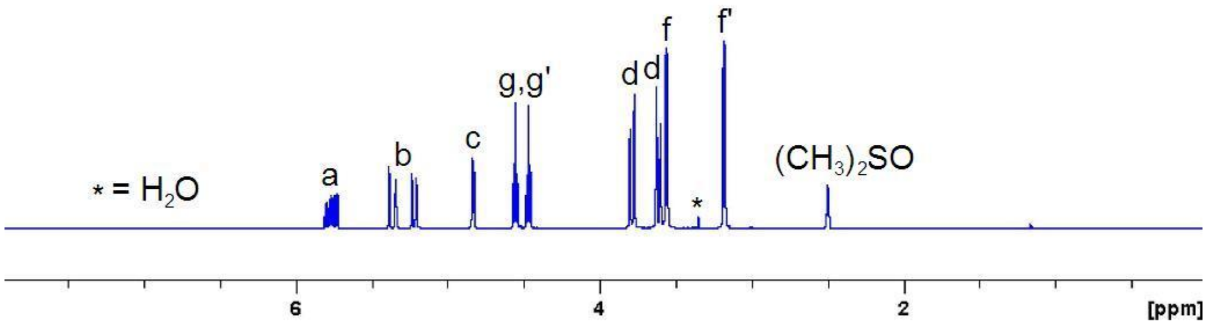

Figure S37. ^1H NMR spectrum of (2-vinyl-1,3-dioxane-5,5-diyl)dimethanol in $(CD_3)_2SO$ (400 MHz, 298 K).

(ii) Synthesis of 9-vinyl-2,4,8,10-tetraoxaspiro[5.5]undecan-3-one (VDC) ***15***

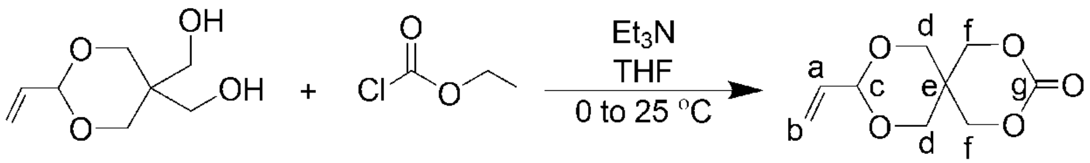

In a round bottom flask (1 L), (2-vinyl-1,3-dioxane-5,5-diyl)dimethanol (20 g, 0.115 mol, 1 eq) and ethylchloroformate (31 g, 0.288 mol, 2.5 eq) was dissolved in THF (600 mL). The solution was cooled in an ice bath and then Et_3N (29 g, 0.288 mol, 2.5 eq) in THF (100 mL)

was added dropwise. After complete addition the ice bath was removed and the mixture stirred for a further 3 h. The white precipitate was separated by filtration and the solvent removed under reduced pressure to yield an off-white solid. This was then dissolved in CH_2Cl_2 and washed water (2 × 50 mL) before being dried with $MgSO_4$. The solvent was removed and the crude product recrystallized in CH_2Cl_2/hexane to yield a white solid (17.0 g, 74%).

^{1}H NMR (400 MHz, $CDCl_3$, 298 K): δ = 5.78 (ddd, $^3J_{H,H}$ = 17.4, 10.7, 4.5 Hz, 1H, H_a), 5.43 (d, $^3J_{H,H}$ = 17.4 Hz, 1H , H_b), 5.30 (d, $^3J_{H,H}$ = 10.7 Hz, 1H, H_b), 4.88 (d, $^3J_{H,H}$ = 4.5 Hz, 1H, H_c), 4.55 (s, 2H, H_f), 4.02 (d, $^3J_{H,H}$ = 12.0 Hz, 2H, H_d), 3.96 (s, 2H, H_f), 3.65 (d, $^3J_{H,H}$ = 12.0 Hz, 2H, H_d). ^{13}C{^{1}H} NMR (100 MHz, $CDCl_3$, 298 K): δ = 148.0 (C_g), 133.4 (C_a), 119.6 (C_b), 101.4 (C_c), 71.3 (C_f), 70.3 (C_f), 68.8 (C_d), 31.5 (C_e). Anal Calcd for $C_9H_{12}O_5$: C 54.00; H 6.04 %. Found: C 53.83; H 6.02 %. HRMS Calcd for $C_9H_{12}O_5Na$: 223.0577; found: 223.0573. Mp: 118-119 °C.

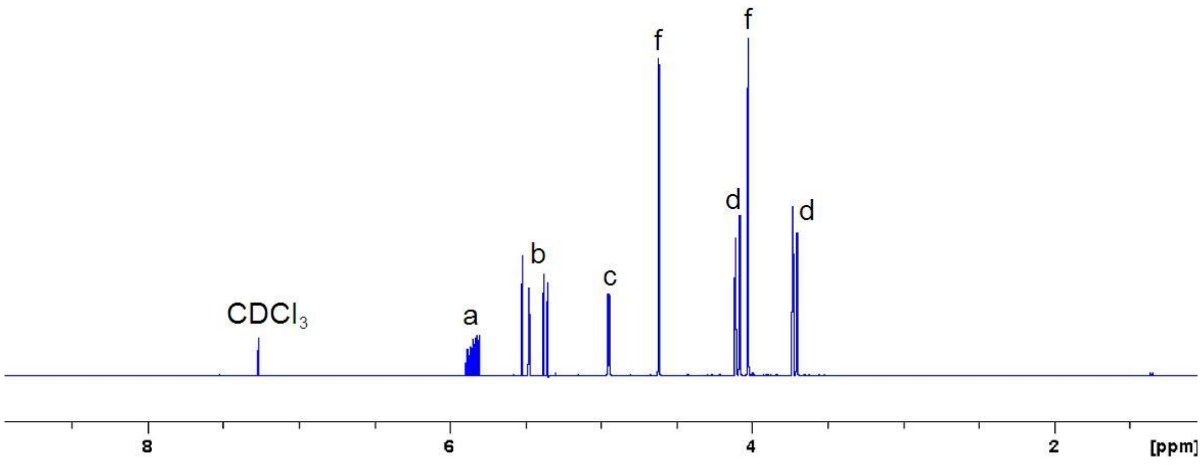

Figure S38. ^{1}H NMR spectrum of VDC in $CDCl_3$ (400 MHz, 298 K).

(iii) Synthesis of end-capped PVDC ***P15***

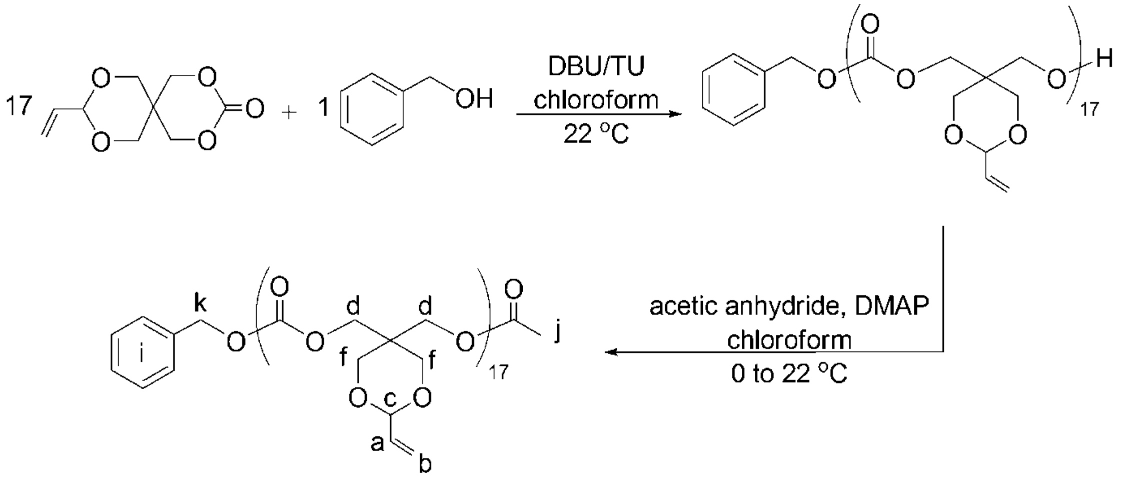

In a glovebox, a solution of benzyl alcohol (0.027 g, 0.250 mmol, 1 eq), 1-(3,5-bis(trifluoromethyl)phenyl)-3-cyclohexylthiourea (TU) (0.0925 g, 0.250 mmol, 1 eq) and 1,8-diazabicycloundec-7-ene (DBU) (0.0076 g, 0.050 mmol, 0.2 eq) in $CHCl_3$ (4 mL) was added to a vial containing VDC (1.0 g, 0.005 mol, 20 eq) dissolved in $CHCl_3$ (6 mL). The solution was stirred for 1.5 h then removed from the glovebox and passed through a plug of silica (eluent: CH_2Cl_2, diethyl ether and ethyl acetate respectively). The ethyl acetate fraction was then precipitated into hexanes to yield PVDC as a solid which was then used directly in the subsequent end-capping process.

In a vial, PVDC (1.0 g, 0.285 mmol, 1 eq) was dissolved in $CHCl_3$ (10 mL). Acetic anhydride (54 µl, 0.570 mmol, 2 eq) was then added followed by DMAP (0.00035 g, 0.003 mmol, 0.01 eq). The reaction was stirred overnight at room temperature and then precipitated into hexanes. The resulting solid polymer was then washed with diethyl ether and dried under vacuum.

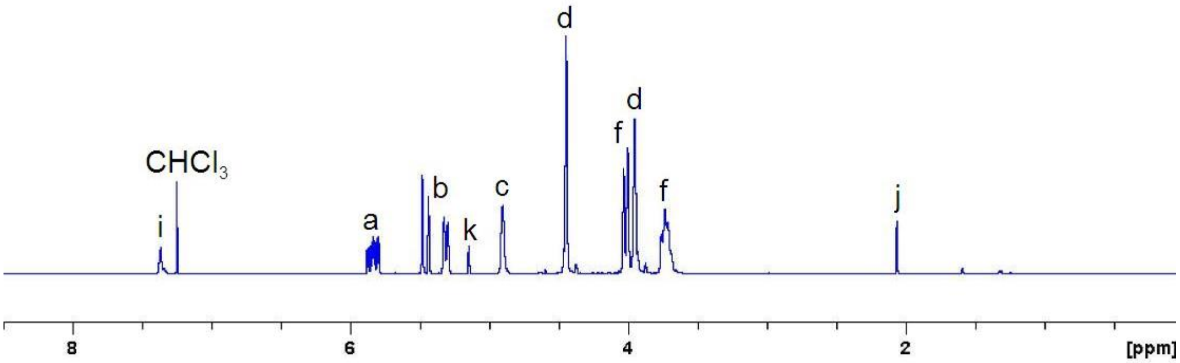

Figure S39. ^{1}H NMR spectrum of end-capped PVDC in $CDCl_3$ (400 MHz, 298 K).

Characterization of **PVDC P15**

^{1}H NMR (400 MHz, $CDCl_3$, 298 K): δ = 7.38 (br m, end-group 5H, H_i), 5.85 (br m, 1H, H_a), 5.55-5.25 (br m, 2H, H_b), 5.16 (s, end-group 2H, H_k), 4.91 (br m, 1H, H_c), 4.46 (br s, 2H, H_d), 4.03 (br m, 2H, H_f), 3.96 (br s , 2H, H_d), 3.74 (br m, 2H, H_f), 2.08 (br s, end-group 3H, H_j). SEC ($CHCl_3$, PS standards) M_n = 5.4 kDa, $Đ_M$ = 1.10.

(iv) Functionalization of PVDC with alcohols

In the glovebox, **PVDC** (50 mg, 0.25 mmol of vinylacetal functionality, DP 17 (^{1}H NMR), M_n = 5.4 kDa ($CHCl_3$ GPC)) and 1-hexanol (32 mg, 0.31 mmol) were dissolved in 1,4-dioxane (0.25 mL). The resulting solution was then added into a Schlenk tube along with catalyst **2** (1.4 mg, 0.0015 mmol, 0.6 mol % relative to total vinylacetal moieties). The reaction vessel was then subsequently sealed, taken out of the glovebox and heated at 45 °C for 12 h. The functionalized polymer was then precipitated into hexane to give **PVDC$_{OE}$** as an off-white gum (62 mg, 83 % yield).

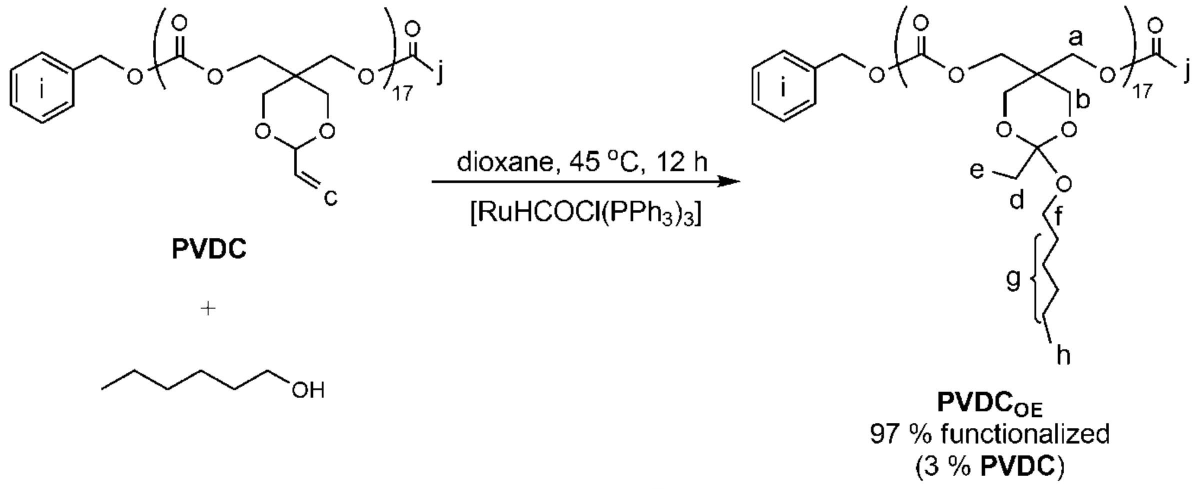

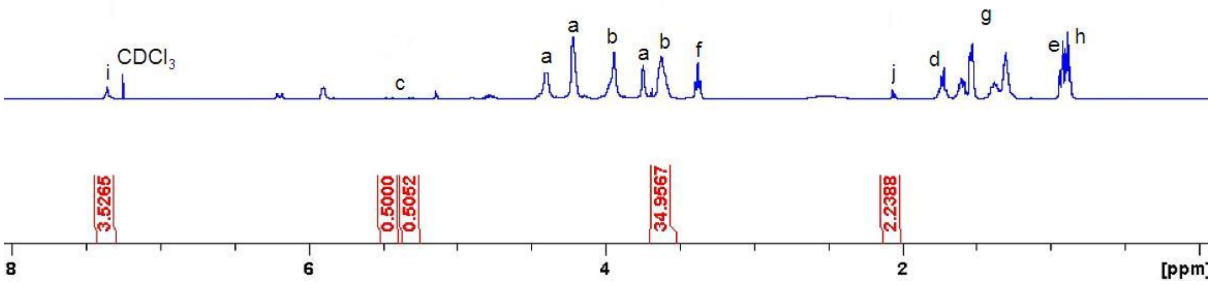

Figure S40. ^{1}H NMR spectrum of **PVDC$_{OE}$** in $CDCl_3$ (400 MHz, 298 K).

Characterization of **PVDC$_{OE}$**

^{1}H NMR (400 MHz, $CDCl_3$, 298 K): δ = 7.36 (m, end-group 5H, H_i), 5.51-5.28 (br m, 2H, H_c), 4.40 (br m, 1H, H_a), 4.22 (br m, 2H, H_a), 3.95 (br m, 2H, H_b), 3.75 (br m, 1H, H_a), 3.63 (br m , 2H, H_b), 3.38 (br t, $^3J_{H,H}$ = 6.7 Hz, 2H, H_f), 2.07 (br s, end-group 3H, H_j), 1.73 (br q, $^3J_{H,H}$ = 6.7 Hz, 2H, H_d), 1.65-1.23 (br m, *8*H, H_g), 0.92 (t, $^3J_{H,H}$ = 6.8 Hz, 3H, H_e), 0.88 (t, $^3J_{H,H}$ = 7.2 Hz, 3H, H_h). SEC ($CHCl_3$, PS standards) M_n = 5.2 kDa, $Đ_M$ = 1.22.

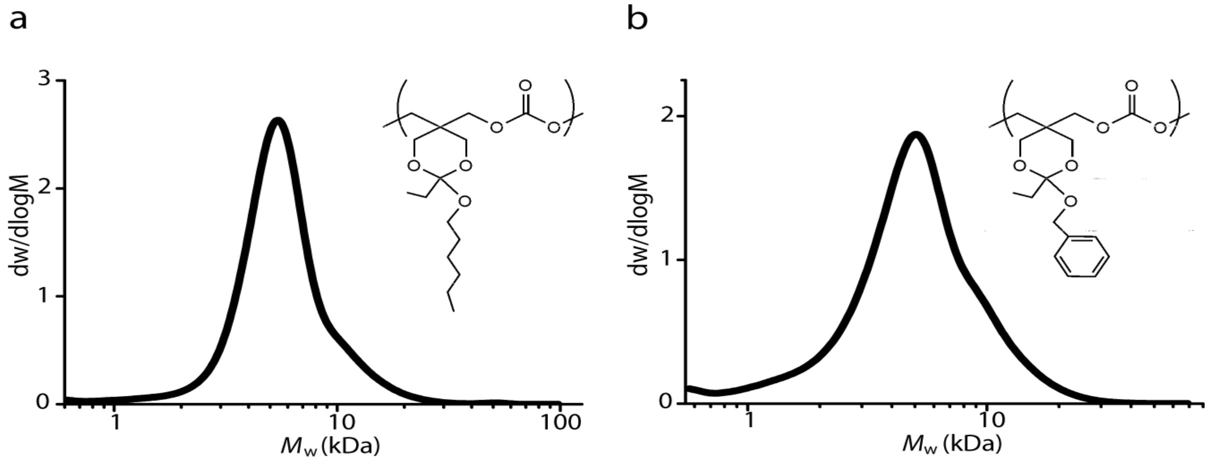

Figure S41. SEC chromatograms of **PVDC$_{OE}$** ($CHCl_3$ eluent, PS standards) functionalized with (a) 1-hexanol (b) benzylalcohol.

3. References

(1) Pratt, R. C.; Lohmeijer, B. G. G.; Long, D. A.; Lundberg, P. N. P.; Dove, A. P.; Li, H.; Wade, C. G.; Waymouth, R. M.; Hedrick, J. L. *Macromolecules* **2006**, *39*, 7863.

(2) Ahmad, N.; Levison, J. J.; Robinson, S. D.; Uttley, M. F. *Inorg. Synth.* **1974**, *15*, 45.

(3) Calabrese, J.; Cushing Jr., M. A.; Ittel, S. D. *Inorg. Chem.* **1988**, *27*, 867.

(4) Crivello, J. V.; Malik, R.; Lai, Y.-L. *J. Polym. Sci. A Polym. Chem.* **1996**, *34*, 3091.

(5) Heller, J.; Barr, J.; Ng, S. Y.; Abdellauoi, K. S.; Gurny, R. *Adv. Drug Deliv. Rev.* **2002**, *54*, 1015.

(6) Fischer, R. F.; Smith, C. W. *J. Org. Chem.* **1960**, *25*, 319.
